# The social transmission of empathy relies on observational reinforcement learning

**DOI:** 10.1073/pnas.2313073121

**Published:** 2024-02-21

**Authors:** Yuqing Zhou, Shihui Han, Pyungwon Kang, Philippe N. Tobler, Grit Hein

**Affiliations:** ^a^ Key Laboratory of Behavioral Science, Institute of Psychology, Chinese Academy of Sciences, Beijing 100101, China; ^b^ Translational Social Neuroscience Unit, Department of Psychiatry, Psychosomatics, and Psychotherapy, University of Würzburg, Würzburg 97080, Germany; ^c^ School of Psychological and Cognitive Sciences, Beijing Key Laboratory of Behavior and Mental Health, PKU-IDG/McGovern Institute for Brain Research, Peking University, Beijing 100871, China; ^d^ Department of Economics and Laboratory for Social and Neural Systems Research, University of Zurich and Neuroscience Center Zurich, University of Zurich and Swiss Federal Institute of Technology Zurich, Zurich CH-8006, Switzerland

**Keywords:** empathy, observational learning, fMRI

## Abstract

Theories of moral development propose that empathy is transmitted across individuals. However, the mechanisms through which empathy is socially transmitted remain unclear. Here, we combine computational learning models and functional MRI to investigate whether, and if so, how empathic and non-empathic responses observed in others affect the empathy of female observers. The results of three independent studies showed that watching empathic or non-empathic responses generates a learning signal that respectively increases or decreases empathy ratings of the observer. A fourth study revealed that the learning-related transmission of empathy is stronger when observing human rather than computer demonstrators. Finally, we show that the social transmission of empathy alters empathy-related responses in the anterior insula, i.e., the same region that correlated with empathy baseline ratings, as well as its functional connectivity with the temporoparietal junction. Together, our findings provide a computational and neural mechanism for the social transmission of empathy that accounts for changes in individual empathic responses in empathic and non-empathic social environments.

## Significance

Humans learn from observing others. Here, we show that such observational learning processes influence the extent to which a person empathizes with the pain of another individual. In our study, female participants observed empathic or non-empathic reactions of others and later provided empathy ratings themselves. Observing empathic reactions of others resulted in an increase, whereas observing non-empathic reaction resulted in a decrease of participants’ homegrown empathy. These changes can be explained by observational reinforcement learning and are reflected by the neural processing of empathy in the anterior insula and its functional connectivity to the temporoparietal junction. Our findings show that empathy can be socially transmitted and observational learning can explain the plasticity of empathic reactions in different social environments.

Empathy—the ability to share the feelings and thoughts of others—can spread across individuals (1). Supporting this notion, there is evidence that self-reported empathy increases if empathy is highly valued by others (2, 3), and when watching empathic responses of others (4). However, these proofs of principle were unable to elucidate the mechanisms through which empathy is socially transmitted.

An influential but untested theory suggests that the social transmission of empathy is based on a learning process that is triggered by observing the empathic reactions of others [“empathic conditioning”(5)]. According to observational learning theory (6), individuals learn from the differences between empathic responses they observe in others and the empathic response they expected to see in others. The mismatch between the observed and expected responses generates so-called observational prediction errors that are known to drive learning-related changes in the actions of the observer (7–9). Here, we investigate whether humans can learn to increase or decrease empathy by observing that others show more or less empathy than predicted.

Neurally, observational learning signals have been associated with activation of the mirror neuron system, including the dorsolateral prefrontal cortex (dlPFC), and premotor cortex, as well as the mentalizing network, including the temporoparietal junction (TPJ), dorsal medial prefrontal cortex (dmPFC), and anterior temporal lobe (ATL) (7–11). Using brain stimulation and computational modeling, a recent study suggested that disruption of the left TPJ weakens participants’ choice adjustment when confronted with dissenting information from others (12). A possible interpretation of this finding is that reduced TPJ activation results in reduced social influence on learning.

So far, learning based on observational prediction errors has been associated with the social transmission of fear (13–15), value-based decision making (7, 8, 16) and the propensity to take or avoid risks (9). Moreover, investigating the social influence on first-hand pain experience, neuroimaging studies showed changes in participants’ pain ratings when they observed pain ratings of others, which were related to changes of neural responses in frontal and parietal brain regions (17) as well as in the anterior insula (AI) (18). However, it remained unclear whether, and if so, how observing empathic reactions to the pain of others affects learning of empathic responses in the observer.

To address this question, we developed an observational-learning-of-empathy paradigm that we applied in four independent studies, combined with computational modeling. The first study (Study 1) was a functional MRI (fMRI) study. The behavioral results of Study 1 were substantiated by the results of a non-social control study (Study 2) and replicated in two independent behavioral studies (Studies 3 and 4).

**Fig. 1.**
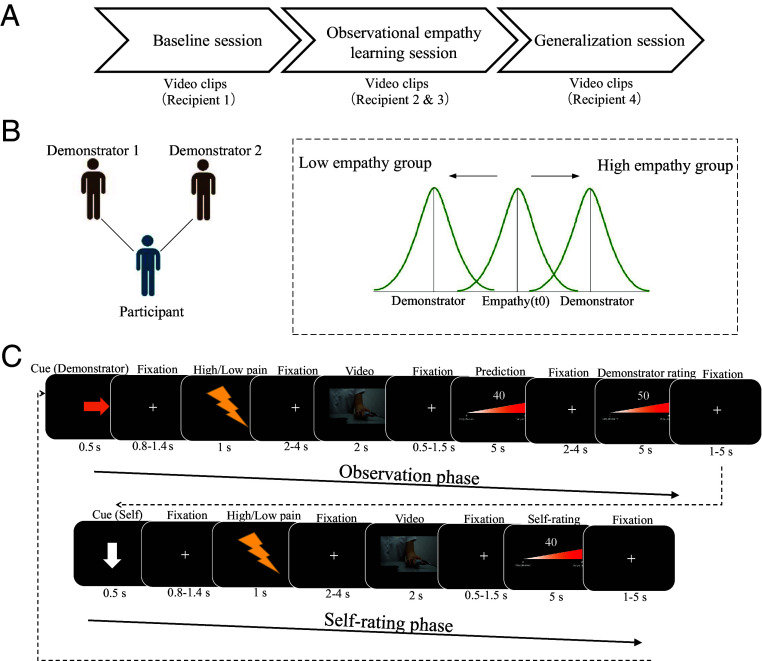
Experimental setup. (*A*) The main experiment consisted of the baseline session, observational empathy learning session, and the generalization session. In each session, participants viewed video clips of different recipients receiving electrical stimulation. The mean pain intensity ratings of recipients were comparable across recipients as indicated by a pilot stimulus validation study (*SI Appendix*, Fig. S1). (*B*) During the observational learning session, participants observed ratings of two demonstrators (Studies 1, 3, and 4: human demonstrators; Study 2: computer demonstrators). The ratings of these demonstrators were generated by a pre-defined algorithm, based on the participant’s empathy ratings in the baseline session (Empathy(t0)) as well as the experimental group the participant was assigned to (i.e., high empathy or low empathy group). (*C*) Example trial of the observational-learning-of-empathy task. Each trial started with an observation phase, followed by a self-rating phase. In the observation phase, the participants observed the ratings of another person (demonstrator) who watched and reacted to the painful stimulation inflicted on a recipient. The observation phase started with an arrow, followed by a lightning bolt cue that indicated the intensity of the recipient’s pain (bright color indicating painful, dark color indicating non-painful stimulation) and a video showing the recipient receiving the respective stimulation. Participants were asked to predict the ratings of the demonstrator for this specific video (Studies 1–3, in Study 4 the prediction screen was replaced by a blank screen) on a scale from zero (predicting that the demonstrator would feel nothing when seeing the other in pain) to hundred (predicting that the demonstrator would feel extremely bad when seeing the other in pain). At the end of the observation phase, the actual rating of the demonstrator was shown. The self-rating phase started with an arrow pointing to the participant. Next, they viewed the cue indicating the intensity of the recipient’s stimulation, watched the video showing the stimulation of the same recipient as in the observation phase, and rated how they felt after seeing the stimulation of the recipient (from 0—not feeling anything, to 100—feeling extremely bad). The trial structure of the non-social control study (Study 2) was identical, except that we presented computer-generated ratings in the observation phase.

All studies consisted of three parts: a baseline session in which we assessed participants’ empathy ratings independently of any experimental manipulation, an observational empathy learning session, and a generalization session that aimed to test whether potential learning-related changes in empathy ratings generalize to individuals that were not part of the learning session (Fig. 1*A*). In the baseline and the generalization session, participants rated their empathy when observing videos showing painful or non-painful stimulation in others. In the observational empathy learning session, participants witnessed the reactions of a demonstrator to the pain of a recipient and were randomly assigned to two groups, a high and a low empathy group. In the high empathy group, participants observed strong empathic reactions whereas in the low empathy group, participants observed weak empathic reactions to the same pain inflicted on the recipient. In the high empathy group, the demonstrator’s ratings of the recipient’s pain were consistently higher than the participant’s baseline ratings, indicating a stronger empathic reaction than the participant’s empathy baseline. In the low empathy group, the demonstrators’ ratings of the recipient’s pain were consistently lower than the participant’s baseline ratings, indicating a weaker empathic reaction compared to the participant (Fig. 1*B*, see *Materials and Methods* for details). In three of the studies, the observed ratings reflected the reactions of a human demonstrator (Studies 1, 3, and 4). In a non-social control study, the observed ratings were from a computer (Study 2). After observing high or low empathic reactions, participants rated how they themselves felt when watching pain in the recipient (Fig. 1*C*).

We hypothesized that observing others would increase the observer’s empathy (as measured by ratings) in the high empathy group and decrease it in the low empathy group. The change in empathy ratings should be driven by learning signals, specifically, observational prediction errors, referring to the discrepancy between the predicted and observed empathy ratings. If the change in empathy is specific to the observational learning from another human, the learning-related changes in empathy ratings should be stronger after observing empathy ratings generated by human demonstrators (Studies 1, 3, and 4) compared to computer-generated ratings (Study 2).

On the neural level, learning others’ empathy responses might be related to activations in the brain regions that were shown to be involved in observational learning and the processing of social influence, including the dlPFC and dmPFC, the premotor cortex (7, 8, 10, 11), and the TPJ (12, 16). Inspired by previous evidence showing that learning from own experiences about others changes empathy-related responses in the AI (19), we further hypothesized that the learning-related changes in empathy may alter the interaction between regions encoding the observational learning signals and the AI.

## Results

### Manipulation Checks Across Studies.

One would expect that participants’ emotion ratings when observing the pain of others would relate to trait empathy. To test this, we used a regression model with the average ratings in the baseline rating session for painful videos of all studies as dependent variable and participants score on the Interpersonal Reactivity Index [IRI, (20)], study (Studies 1–4), and study × IRI score as predictors. This analysis revealed a significant effect of IRI score (*β =*0.226*, t* = 3.34, *P* = 0.001), but no significant effects of study (*β =*0.029*, t* = 0.432, *P* = 0.666) and study × IRI score (*β =* 0.042*, t* = 0.624, *P* = 0.534), indicating that across studies, the emotion ratings elicited by watching the painful stimulation of recipients were similarly related to trait empathy.

As a second manipulation check, we assessed the expectation that observing high and low empathic responses should differently change participants’ impressions of the demonstrator. To test this, we used the pre- and post-learning impression scores (19, 21) of Studies 1, 3, and 4 (i.e., the studies including human demonstrators) as dependent variable and study (study 1/3/4), group (high, low empathy) and time (pre-, post-learning experiment) as predictors. We found a significant group × time interaction (*F =* 18.51*, P =* 0.01), which occurred similarly for all studies with human demonstrators (*F =* 0.01*, P =* 0.99). While participants’ impressions toward the demonstrators did not differ between the high and low empathy groups before the experiment (*β =* 0.01*, t =* 0.15*, P =* 0.88), their impression ratings toward the demonstrators were more positive in the high compared to the low empathy group after the experiment (*β =* 0.39*, t =* 5.15*, P <* 0.001). This demonstrates that our experimental manipulation (observing empathic vs non-empathic responses) had an influence on how participants perceived the demonstrators, thus, validating the social manipulation.

### Results of the fMRI Study.

#### *Regression (model-independent) analyses of behavior*.

**Fig. 2.**
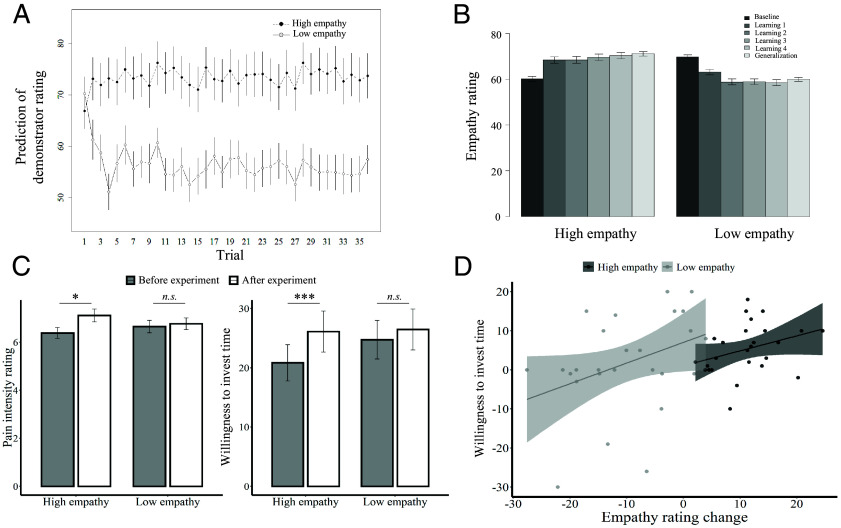
Observation-induced changes in predictions, empathy ratings, and willingness to spend time to help after learning. (*A*) Predictions of demonstrator ratings in the observation phase (mean across participants) increased in high empathy (black dots) but decreased in low empathy (white dots) groups across trials. (*B*) Averaged empathy ratings in each session of the experiment show an increase in the high empathy group, and a decrease in the low empathy group. (*C*) Average pain intensity ratings and willingness to spend time to help the recipient increased in the high empathy group after learning. (*D*) In both groups, the change in empathy rating from the baseline to the generalization session was related to participants’ willingness to spend time to help the recipient.

In the observation phase, participants predicted the empathy ratings of the demonstrator. Entering these prediction ratings as dependent variable in a linear mixed model (LMM) with group (high empathy, low empathy), trial number and group × trial number as predictors revealed a significant group × trial number interaction (*χ*^2^(1) = 26.04, *P* < 0.001), indicating that participants expected increasing empathy ratings of the demonstrators in the high (*χ*^2^(1) = 3.88, *P* = 0.05) and decreasing empathy ratings in the low empathy (*χ*^2^(1) = 27.58, *P* < 0.001) group (Fig. 2*A*).

Next, we analyzed participants’ own empathy ratings from the self-rating phase. An LMM with group (high empathy, low empathy), trial number and group × trial number as predictors revealed a significant group × trial number interaction (*χ*^2^(1) = 39.57, *P* < 0.001), indicating an increase in participants’ empathy ratings in the high (*χ*^2^(1) = 5.44, *P* = 0.02) and a decrease in empathy ratings in the low empathy group (*χ*^2^(1) = 41.60, *P* < 0.001). Similarly, an LMM with group (high empathy, low empathy), session (baseline, observational empathy learning (1–4) and generalization, coded as 0–5 respectively), and group × session as predictors and participants’ empathy ratings in the respective session as the dependent variable showed a significant group × session interaction (*χ*^2^(1) = 116.5, *P* < 0.001, Fig. 2*B*). There was a trend toward a difference in baseline empathy ratings between the low and high empathy group (*t*(50) = 1.87, *P* = 0.067). Separate analyses then showed a significant increase in empathy ratings across sessions in the high empathy group (*χ*^2^(1) = 101.3, *P* < 0.001, Fig. 2*B*), and a significant decrease in empathy ratings across sessions in the low empathy group (*χ*^2^(1) = 40.40, *P* < 0.001, Fig. 2*B*).

The observed changes in participants’ empathy ratings might be driven by social desirability and the wish to conform with the ratings of the demonstrators, and influenced by empathy baseline ratings. To evaluate the influence of social desirability and conformity on the change in empathy ratings during learning, we calculated the individual scores measured from social desirability [SDS-17, (22)] and conformity scales (23) for the high and low empathy group separately. We then conducted a regression analysis with the change in empathy ratings between baseline and generalization sessions as dependent variables, and the individual scores on social desirability and conformity scales as predictors. We also included the averaged baseline empathy ratings as predictor to check whether individual differences in empathy baseline ratings account for group differences in subsequent empathy changes. The analyses revealed no significant effects (*SI Appendix*, Table S1, *ps* > 0.31), rendering the possibility unlikely that the individual changes in empathy ratings were driven by individual differences in social desirability and conformity or by baseline differences in empathy. Together, these results show that participants shifted their empathy ratings toward the ratings of the demonstrators, that these changes could not be explained by social desirability and that they were preserved even when participants were no longer presented the demonstrators’ ratings (i.e., generalization session).

Participants also reported how much pain they thought the person in the video clip was experiencing and how much time they were willing to spend in order to help the pain recipient before and after the experiment. Consistent with the change in empathy ratings, participants in the high empathy group evaluated the intensity of the pain experienced by the recipient as significantly stronger (M = 7.1 vs. 6.4, *t*(25) = 2.71, *P* = 0.01, Fig. 2*C*) and were willing to spend more time to help the recipient after learning (M = 26.1 vs. 20.8 min, *t*(25) = 4.16, *P* < 0.001, Fig. 2*C*) compared to before learning. In contrast, there were no such learning-related changes in the low empathy group (pain intensity: M = 6.8 vs. 6.7, *t*(25) = 0.46, *P* = 0.65; prosocial tendency: M = 26.5 vs. 24.7 min, *t*(25) = 0.7, *P* = 0.49, Fig. 2*C*). Finally, in both groups, the difference in empathy ratings between the baseline and the generalization session predicted the individual pre-to-post difference in the willingness to spend time in order to help the recipient (high and low empathy group combined: *ρ* = 0.345, *P* = 0.012; high empathy group only, *ρ* = 0.407, *P* = 0.039; low empathy group only, *ρ* = 0.392, *P* = 0.048, Fig. 2*D*). Together, these results suggest that observing the empathic reactions of the demonstrators changed the predictions and empathy ratings of the observer. Moreover, changes in empathy ratings influenced participants’ willingness to invest time in order to help the recipient.

#### *Reinforcement learning model-based analyses of behavior*.

**Fig. 3.**
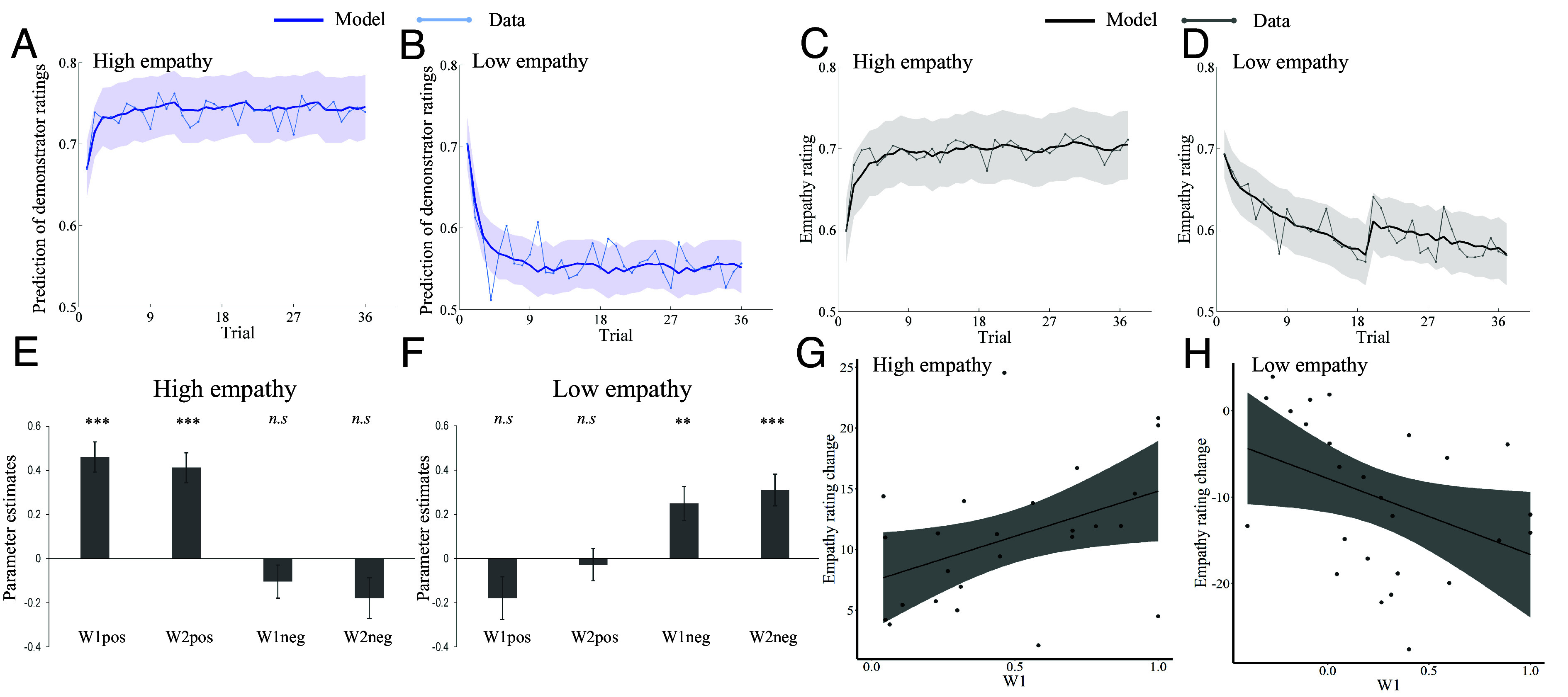
Computational models explain predictions and changes in empathy ratings. (*A* and *B*) Predictions of human demonstrator ratings (light blue line) increased in the high empathy and decreased in the low empathy group, and our learning model explained these changes (dark blue line, shaded area represents ±1 SE). (*C* and *D*) Trial-by-trial empathy ratings (light gray) and corresponding model estimates (dark gray, shaded area represents ±1 SE) for the high and the low empathy groups. The model estimates illustrate the best-fitting model. (*E* and *F*) Weight parameters for high (*E*) and low (*F*) empathy groups. The weight parameters for positive (negative) observational prediction errors were significantly larger than zero in high (low) empathy groups. (*G* and *H*) The weight parameters in the first half of the learning experiment (i.e., *W1*) significantly correlated with the change in empathy ratings across participants for both high and low empathy groups.

Having demonstrated that participants changed their empathy ratings after observing the ratings of others, we next sought to examine the computational mechanisms supporting these changes. We first modeled participants’ trial-by-trial predictions of demonstrator ratings using a Rescorla–Wagner reinforcement-learning model (24). The model fitted the data adequately for both the high and low empathy groups [*r*^2^ (mean ± SD) = 0.22 ± 0.21 and 0.27 ± 0.21; Fig. 3 *A* and *B*, see *Materials and Methods* for details]. The estimated learning rate was comparable for high and low empathy groups [α: *t*(50) = −0.179, *P* = 0.859, 95% CI = [−0.08, 0.07], *SI Appendix*, Table S2], suggesting that participants learned to predict the ratings of empathic and non-empathic demonstrators similarly well.

Next, we modeled trial-by-trial update of participants’ empathy ratings as a linear function of the cumulative impact of observational prediction errors (21, 25, 26), as estimated by the reinforcement learning (RL) model. Bayesian model selection was used to identify the model that was most probable to generate the data, based on Laplace approximation (see *Materials and Methods* for details). The winning model (*SI Appendix*, Eq. **S5**, Model 3, XP: 1) successfully captured dynamic changes of empathy ratings at the individual level for both high (*r^2^* = 0.19 ± 0.14) and low (*r*^2^ = 0.24 ± 0.12) empathy groups (Fig. 3*D*). The winning model (*SI Appendix*, Eq. **S5**, Model 3) assumes that the empathy ratings of participants for each trial *t* are driven by the time-discounted sum of previous observational prediction errors. It considers the empathy ratings in the first half and second half of the learning session separately and adds up separately modeled positive and negative observational prediction errors.

In this model, two parameters (*W1* and *W2*) capture the magnitude (weight) of the influence of observational prediction errors on changes in participants’ empathy ratings in the first and second half of the observational learning session. The weights are separated by the sign of the observational prediction errors (indicated by the pos/neg subscript). A larger *W* corresponds to a stronger influence of observational prediction errors on participants’ empathy ratings. The discount parameter *γ* (0 ≤ *γ* ≤ 1) captures an exponentially decaying influence of previous observational prediction errors over time, such that more recent observational prediction errors have a greater impact on the changes in empathy ratings than earlier observational prediction errors. If *γ* is close to one, all preceding observational prediction errors receive the same weight, and if it is close to zero, only the last observational prediction error leads to subsequent changes in empathy ratings.

We fitted the empathy ratings separately for the high and the low empathy group. For the high empathy group, the weight parameters on positive observational prediction errors were significantly larger than zero [*W1_pos_: t*(25) = 6.79, *P* < 0.001; *W2_pos_: t*(25) = 6.12, *P* < 0.001, Fig. 3*E* and *SI Appendix*, Table S2], whereas the weight parameters on negative observational prediction errors were not different from zero [*ts* > −1.94, *ps* > 0.06, Fig. 3*E* and *SI Appendix*, Table S2]. By contrast, the weight parameters on negative observational prediction errors were significantly larger than zero in the low empathy group [*W1_neg_: t*(25) = 3.23, *P* = 0.003; *W2_neg_: t*(25) = 4.34, *P* < 0.001, Fig. 3*F*, *SI Appendix*, Table S2], whereas the weight parameters on positive observational prediction errors were not different from zero (*ts* > −1.87, *ps* > 0.07, Fig. 3*F* and *SI Appendix*, Table S2). These results suggest that participants in the high and low empathy groups were predominantly influenced by the positive and negative observational prediction errors, respectively. We also checked the relationships between the weight parameters and the individual scores on social desirability and conformity scales. The analyses revealed no significant effects (*ps* > 0.16), providing little support for the notion that individual weights on observational prediction errors were influenced by individual differences in social desirability and conformity.

Next, we correlated the weight parameters with the change in empathy ratings across participants. The respective weight parameters in the first half of the learning session (i.e., *W1_pos_* for the high empathy group and *W1_neg_* for the low empathy group) were significantly associated with the increase in empathy rating in the high empathy group (*ρ* = 0.39, *P* = 0.047, Fig. 3*G*) and the decrease in empathy rating in the low empathy group (*ρ* = −0.50, *P* = 0.009, Fig. 3*H*), whereas the weight parameters in the second learning session were not (*ps* > 0.154). These results suggest that the weight of the observational prediction errors in the first half of the learning experiment majorly drives the overall changes in empathy ratings.

#### *Neuroimaging results*.

**Fig. 4.**
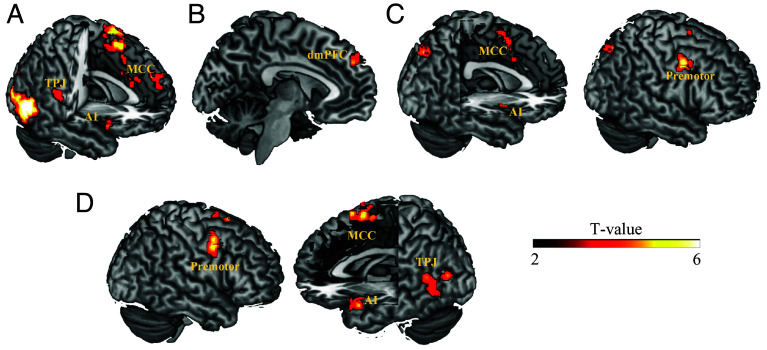
Neuroimaging results. (*A*) Neural responses associated with trial-by-trial empathy ratings in the baseline session. (*B*) Neural representation of observational prediction errors in the high empathy group. (*C*) Neural representation of observational prediction errors in the low empathy group. (*D*) Regions encoding observational prediction errors differently between high and low empathy groups. Significant clusters were identified by combining a voxel-level threshold of *P* < 0.001 (uncorrected) and a cluster-level threshold of *P* < 0.05, *FWE corrected* across the whole brain. Display threshold at *P*_uncorrected_ < 0.001; AI = anterior insula, MCC = mid cingulate cortex, TPJ = temporoparietal junction; dmPFC = dorsal medial prefrontal cortex; premotor = premotor cortex.

Our fMRI data analyses focused on the neural mechanisms underlying the observational learning of empathy. As a manipulation check, we first examined the neural signals that significantly correlated with the trial-by-trial empathy rating when viewing the videos in the baseline session (i.e., before learning). Whole-brain analyses across all participants revealed activations in the dorsal medial cingulate (dMCC), bilateral AI, and the bilateral TPJ (Fig. 4*A*), replicating the results of previous neuroimaging studies on the neural basis of empathy for pain (27–32).

Then, we investigated brain regions encoding observational prediction errors as identified by our computational model. Specifically, we regressed trial-by-trial observational prediction errors from the winning model as parametric modulator against neural activity when the ratings of demonstrators were revealed. In the high empathy group, the trial-wise observational prediction errors were related to activation in the dmPFC (Fig. 4*B* and *SI Appendix*, Table S3*A*), such that dmPFC activation was stronger when demonstrator ratings were higher than expected. In the low empathy group, we only observed significant neural responses with the inverse observational prediction errors, reflected by an increase of activation in the bilateral premotor cortex, the medial cingulate cortex (MCC), and the AI when demonstrator ratings were lower than expected (Fig. 4*C* and *SI Appendix*, Table S3*B*).

Next, we compared the neural coding of observational prediction errors between the high and low empathy groups. The results revealed significant group differences in the TPJ, the MCC, premotor cortex, occipital cortex, and AI (extending into the anterior temporal pole) (Fig. 4*D* and *SI Appendix*, Table S3*C*). These regions showed stronger activations when demonstrator ratings were higher than expected in the high empathy group (*ts* > 2.78, *ps* < 0.010). In contrast, in the low empathy group, activations in these regions were stronger when demonstrator ratings were lower than expected (*ts* < −3.56, *ps* < 0.002).

Based on our modeling results, we further tested whether neural regions which differentially encoded observational prediction errors in the observation phase also showed group-related differential connectivities with regions encoding empathy-related activity in the self-rating phase. The strength of this functional coupling should depend on the individual weight given to the observational prediction errors (i.e., the *W1* parameter that accounted for the learning-related changes in empathy ratings). Given that the observational learning network contained several regions, we conducted a multi-region PPI analysis (33, 34), which allows defining multiple seed regions and simultaneously assessing the respective connectivity changes depending on a given variable (here *W1*). We defined the seed regions by the brain regions that showed the strongest differential coding of observation prediction errors between groups (*SI Appendix*, Table S3*C*). We calculated the connectivity strength between each of these seeds and 264 target regions that were defined with an established template (35), and assessed which of these connectivities was modulated by the *W1* parameter. Visualization of the suprathreshold edges revealed that the left TPJ showed the largest number of connectivities that were influenced by the magnitude of *W1*. This result held when we used different threshold values (ranging from 0.001 to 0.05) to identify significant connectivities, indicating the robustness of our results (*SI Appendix*, Fig. S2).

Based on the results of the multi-region PPI, we chose the left TPJ (Fig. 4*D*) as a seed to estimate its connectivity strength with other brain regions when viewing others in pain in the self-rating phase (first-level analysis). We then conducted a second-level analysis with the individual *W1* parameter (*W1_pos_* for the high empathy group and *W1_neg_* for the low empathy group) as a covariate.

**Fig. 5.**
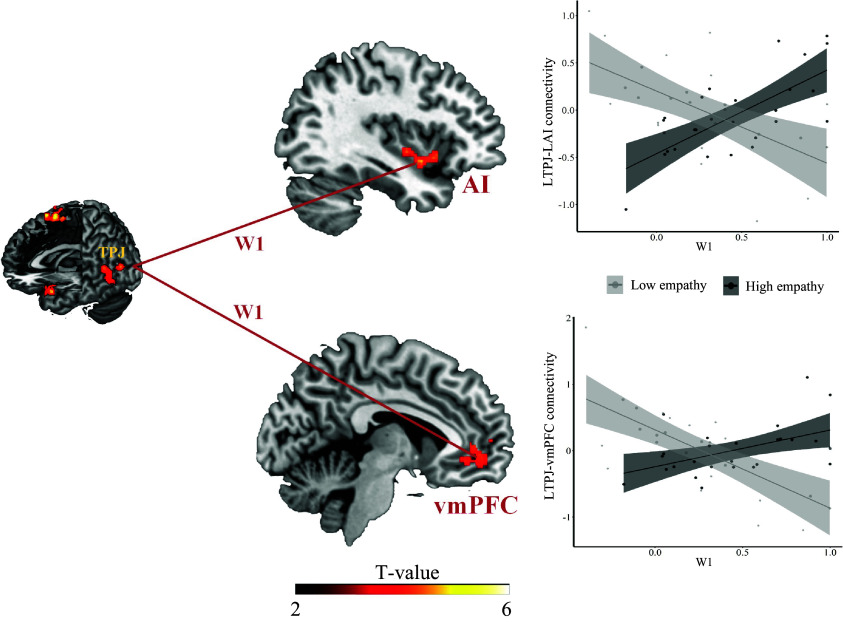
Group-specific impact of weight given to observational prediction errors on functional connectivity. The functional connectivity between the TPJ and AI, and between the TPJ and vmPFC during the self-rating phase correlated with the weights given to observational prediction errors across participants for both high and low empathy groups. Significant clusters were identified by combining a voxel-level threshold of *P* < 0.001 (uncorrected) and a cluster-level threshold of *P* < 0.05, *FWE corrected* across the whole brain. Display threshold at *P*_uncorrected_ < 0.001; TPJ = temporoparietal junction, AI = anterior insula, vmPFC = ventral medial prefrontal cortex.

Whole-brain analysis showed that the individual *W1* parameter modulated the connectivity of the left TPJ with the left AI (MNIxyz: −38/4/−10, Z_stats_= 4.25, *P(cluster-FWE)* = 0.024), and with the vmPFC (MNIxyz: −8/50/−4, Z_stats_= 4.63, *P(cluster-FWE)* < 0.001) differently in high and low empathy groups while participants watched painful videos during the self-rating phase (Fig. 5). Specifically, the more strongly individuals weighted observational prediction errors (i.e., larger *W1* parameters), the weaker the left TPJ-vmPFC coupling in the low empathy group (*r* = −0.70, *P* < 0.001), and the stronger the left TPJ-vmPFC coupling in the high empathy group (*r* = 0.49, *P* = 0.010). Similarly, with increasing *W1* parameter, the coupling between the left TPJ and left AI increased in the high empathy group (*r* = 0.71, *P* < 0.001), and decreased in the low empathy group (*r* = −0.59, *P* = 0.001) (Fig. 5). Importantly, the same AI-region that showed connectivity with the TPJ depending on the strength of the observational learning signal (*W1*) was also significantly correlated with the trial-by-trial empathy ratings in the baseline session (*t*(51) = 2.31, *P* = 0.025), indicating that observational learning changed the communication of the TPJ with an AI region that is involved in the processing of empathy.

To test the specificity of these results, we performed a control analysis in which we estimated the connectivity strength between the left TPJ, and other brain regions when participants watched the painful videos in the observation phase (i.e., not the self-rating phase), and regressed this connectivity against the *W1* parameter in both groups. This analysis revealed no significant group differences in the impact of *W1* on TPJ connectivity even at a lenient threshold (i.e., *P* < 0.05, uncorrected). Thus, the group differentiating effect of the weight given to observational prediction errors on TPJ-AI as well as on TPJ-vmPFC connectivity was specific to the self-rating phase.

**Fig. 6.**
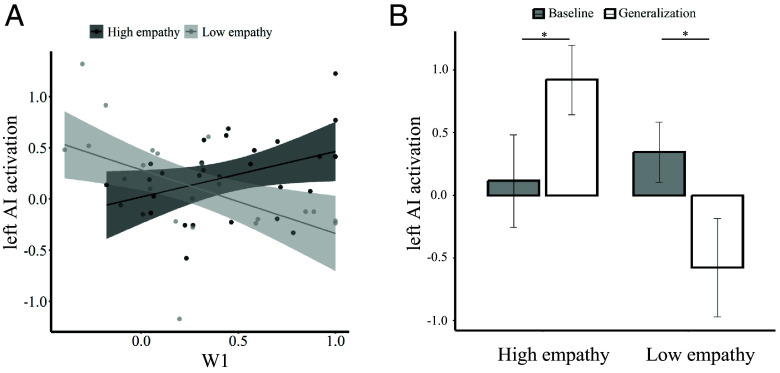
Neural responses in the AI region identified by the PPI analysis (Fig. 5, *Upper* panel). (*A*) The activation of AI correlated with the weights given to observational prediction errors across participants for both high and low empathy groups. (*B*) Participants in the high empathy group showed increased AI activation in generalization compared to baseline session whereas participants in the low empathy group showed the reverse pattern.

To specify the results of the PPI analysis, we tested whether the observed AI region is associated with changes in empathy during the self-rating phase. To do so, we regressed the individual *W1* parameters (*W1_pos_* for the high empathy group and *W1_neg_* for the low empathy group) against the neural activity to the painful videos in the self-rating phase, and calculated the contrast between the high and the low empathy groups. The results showed significant activation in the AI (*P* = 0.021, SVC-FWE corrected). Post hoc comparisons revealed that an increase in *W1* resulted in an increase in AI activation in the high empathy group (*r* = 0.379, *P* = 0.056, Fig. 6*A*), and in a decrease of AI activation in the low empathy group (*r* = −0.514, *P* = 0.007, Fig. 6*A*). As our behavioral results indicated that the learning effects were preserved even when participants were no longer presented with the demonstrators’ ratings (i.e., generalization session), we further compared the neural activations of left AI before (i.e., baseline session) and after (i.e., generalization session) learning between high and low empathy groups. The results showed a significant group (high empathy, low empathy) × session (baseline session, generalization session) interaction (peak = −36/−2/−6, *P* = 0.030, SVC-FWE corrected for the left AI cluster identified in the PPI analysis). Besides, left AI responses were increased in participants in the high empathy group after learning (*t*(25) = 2.18, *P* = 0.039, Fig. 6*B*) and decreased in the low empathy group after learning (*t*(25) = −2.52, *P* = 0.018, Fig. 6*B*). The same analyses in vmPFC did not reveal any significant results (SVC-FWE correction, *ps* > 0.289). Together, these results suggest that the observational learning signals alter empathy-related responses at the neural level.

### Results of the Non-Social Control Study.

The results of our fMRI study demonstrated significant changes in empathy ratings in both high and low empathy groups. The computational model further linked the changes in empathy ratings to the weight given to observational prediction errors. However, it is possible that participants provided higher ratings in the high empathy group compared to the low empathy group only because they were shown larger numbers. Viewing larger or smaller numbers could anchor participants’ responses on these values, thereby creating systematic biases (36). To examine this possibility as well as the extent to which the observational learning effect depends on observing the behavior of human vs. nonhuman computer demonstrators, we investigated observational learning from non-social demonstrators (i.e., from computer-generated ratings) in a non-social control study (Study 2).

**Fig. 7.**
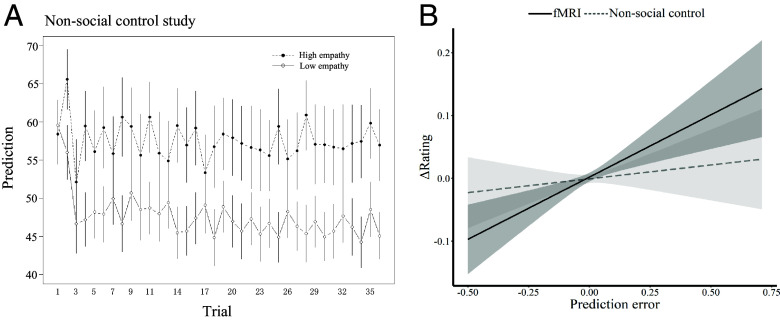
Participants learn, but to a lesser degree, from computer compared to human demonstrators. (*A*) Trial-by-trial prediction ratings. The results showed differential effects in the high and low empathy groups in the non-social control study. (*B*) Study × prediction error interaction. The observational prediction errors shifted participants’ empathy ratings more strongly in the fMRI study (dark gray) compared to the non-social control study (light gray).

We first tested whether participants would learn to predict the observed empathy ratings of the computer demonstrators similarly to participants who learned to predict the observed empathy ratings of human demonstrators. To this end, we conducted an LMM with study (fMRI, non-social control), group (high empathy, low empathy), trial number and group × trial number as predictors, and participants’ predictions of demonstrators’ ratings as the dependent variable. The results revealed a significant group × trial number interaction (*χ*^2^(1) = 8.31, *P* = 0.004). The study × group × trial number interaction was not significant (*χ*^2^(1) = 0.22, *P* = 0.64, Fig. 7*A*), indicating that participants paid attention to the computer-generated ratings and learned to predict them.

Next, we tested whether participants’ empathy ratings were similarly influenced by human and computer demonstrators. As in the analysis of the fMRI study, we first fitted the participants’ predictions of (computer) demonstrators’ ratings using a Rescorla–Wagner reinforcement-learning model (24). The RL model fitted the predictions of computer demonstrators’ ratings adequately (*r^2^* = 0.17 ± 0.19 for the high empathy group, *r^2^* = 0.29 ± 0.22 for the low empathy group), and did not differ from the fMRI study (*t*(50) = 0.996, *P* = 0.324 for the high empathy group; *t*(54) = −0.369, *P* = 0.713 for the low empathy group). We then extracted the trial-wise observational prediction errors from both the fMRI study and the non-social control study and fitted an LMM to directly test the association between trial-wise observational prediction errors and changes in empathy ratings. If participants change their empathy ratings based on the observational prediction errors, we would expect a positive association between trial-wise observational prediction errors and changes in empathy ratings. The LMM included study (fMRI, non-social control), empathy group (high empathy, low empathy), and trial-wise observational prediction errors, as well as their interactions as fixed effects predicting trial-wise changes of empathy ratings. The analysis revealed a significant study × prediction errors interaction (*χ*^2^(1) = 5.34, *P* = 0.021, *SI Appendix*, Table S4 for full statistical results) indicating a stronger relationship between trial-wise prediction errors and changes in empathy ratings in the fMRI study, compared to the control study (Fig. 7*B*). Thus, although participants predicted the ratings of human demonstrators and computer demonstrators similarly well, the observations of the computer influenced the empathy ratings of participants to a lesser extent than the observations of the human demonstrator.

### Results of the Behavioral Replication Studies.

To test the reproducibility of the learning effects observed in the fMRI study, we conducted two additional behavioral studies (Studies 3 and 4), using two independent samples.

#### *Study 3: Behavioral replication*.

In Study 3, we used the identical paradigm as in Study 1, but further minimized the contact to the experimenter to reduce the effect of social desirability. Whereas in Study 1 participants were placed inside the fMRI scanner and thus had some contact to the experimenter, in Study 3 they were seated alone in the behavioral experimental room.

**Fig. 8.**
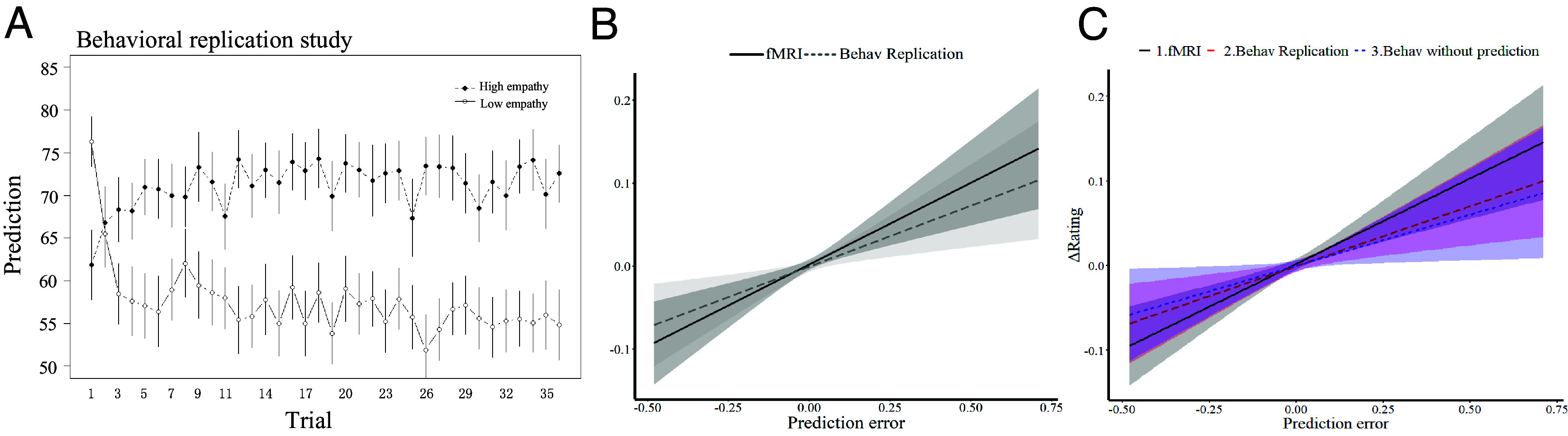
Replication of behavior in the fMRI study. (*A*) Trial-by-trial prediction in the behavioral replication study. The results showed differential effects in the high and low empathy groups. (*B*) Effect of prediction error on changes in empathy ratings in the behavioral replication study (light gray) and the fMRI study (dark gray). The interaction of study and prediction error was not significant, indicating comparable observational learning of empathy in both studies. (*C*) Effect of prediction error on changes in empathy ratings in the behavioral replication study (red), the fMRI study (black), and the behavioral study without predictions (blue). The interaction between study × prediction error was not significant, indicating comparable observational learning of empathy in the three studies.

We first analyzed the predictions from the observational learning phase. To this end, we conducted an LMM with group (high empathy, low empathy), trial number and group × trial number as predictors, and participants’ predictions of ratings as the dependent variable. The results revealed a significant group × trial number interaction (*χ*^2^(1) = 67.4, *P* < 0.001, Fig. 8*A*), indicating that participants expected increasing empathy ratings of the demonstrators in the high (*χ*^2^(1) = 14.37, *P* < 0.001) and decreasing empathy ratings in the low empathy (*χ*^2^(1) = 62.4, *P* < 0.001) group also in this independent sample.

We then tested the participants’ own empathy ratings in the self-rating phase of the observational learning session. The LMM with group (high empathy, low empathy), trial number and group × trial number as predictors, and participants’ empathy ratings as the dependent variable revealed a significant group × trial number interaction (*χ*^2^(1) = 18.56, *P* < 0.001). Replicating the results of the fMRI study, participants showed increasing empathy ratings in the high empathy group (*χ*^2^(1) = 4.46, *P* = 0.03), and decreasing empathy ratings in the low empathy group (*χ*^2^(1) = 16.82, *P* < 0.001) over the course of learning. We also analyzed the empathy ratings of our participants over the whole experiment (i.e., from the baseline session to the generalization session). To this end, we conducted an LMM with group (high empathy, low empathy), session (baseline session, observational empathy learning session 1–4 and generalization session, coded as 0–5 respectively) and group × session as predictors, and participants’ empathy ratings as the dependent variable. Similar to the results of the fMRI study, we found a significant group × session interaction (*χ*^2^(1) = 34.4, *P* < 0.001), with an increase in ratings across sessions in the high empathy group (*χ*^2^(1) = 9.13, *P* = 0.003) and a decrease in ratings across sessions in the low empathy group (*χ*^2^(1) = 37.6, *P* < 0.001). In summary, the prediction data and participants’ own empathy ratings in the behavioral replication study resembled those of the fMRI study.

To test whether these changes in empathy ratings were associated with the observational learning mechanism revealed in Study 1, we first fitted the participants’ predictions using a Rescorla–Wagner reinforcement-learning model (24). The RL model fitted the predictions adequately (*r^2^* = 0.22 ± 0.19 for the high empathy group, *r^2^* = 0.28 ± 0.19 for the low empathy group), and did not differ from the fMRI study (*t*(49) = 0.001, *P* = 0.999 for the high empathy group; *t*(51) = −0.212, *P* = 0.833 for the low empathy group). We then extracted the trial-wise observational prediction errors and associated them with trial-wise changes in empathy ratings in an LMM. The results revealed that trial-wise observational prediction errors positively predicted the trial-wise changes of empathy ratings (*χ*^2^(1) = 25.75, *P* < 0.001, Fig. 8*B*), and similarly well in the high and the low empathy group (*χ*^2^(1) = 0.225, *P* = 0.61).

To compare studies more thoroughly, we also interrogated an additional LMM with study (fMRI, behavioral replication), empathy group (high empathy, low empathy), and trial-wise observational prediction errors, as well as their interaction to predict the trial-wise changes of empathy ratings. The analysis showed that the study × prediction errors interaction effect was not significant (*χ*2(1) = 0.55, *P* = 0.46, Fig. 8*B* and *SI Appendix*, Table S4 for full statistical results), compatible with the notion that participants’ empathy ratings were similarly influenced by the observational prediction errors in the fMRI study and the behavioral replication study. In summary, the behavioral replication study (Study 3) resulted in similar behavior as the fMRI study.

#### *Study 4: Behavioral replication study without prediction*.

The experimental procedure in Study 4 was identical to Studies 1 and 3, except that participants were no longer asked to predict the ratings of the demonstrator. This experimental manipulation was important to rule out an “implicit instruction” effect, i.e., the possibility that in Studies 1 and 3 participants provided similar ratings as the demonstrator because they were instructed to predict the demonstrator’s behavior.

As in the analyses of Study 3, we examined the change of empathy ratings during the experiment and linked the trial-wise observational prediction errors to predict the trial-wise changes of empathy ratings. The results revealed significant differences in participant ratings between high and low empathy groups over time (χ^2^(1) = 20.38, *P* < 0.001) that were comparable to the effects observed in Studies 1 and 3 in which participants had to predict the demonstrator’s ratings (χ^2^(2) = 0.52, *P* = 0.77). In addition, participants’ ratings in Study 4 were similarly influenced by the observational prediction errors as in Study 1 and 3 (χ^2^(2) = 0.59, *P* = 0.75, Fig. 8*C* and *SI Appendix*, Table S4 for statistical details). These findings suggest that participants learn similarly when they are asked to predict or not to predict the demonstrator’s ratings (see *SI Appendix*, *Supplementary Results, Behavioral replication study without prediction*, for details).

## Discussion

The assumption that empathy can be transmitted between individuals forms the basis of influential theories of moral development (1). Here, we provide mechanistic insights into the social transmission of empathy. Confirmed in three independent studies and substantiated by a control study, our results showed that empathy is transmitted by learning from observed empathic reactions of others. The observational learning of empathy can increase or decrease empathy in the observer, depending on the role model the participants learn from. Notably, the learning-related changes in empathy were elicited by observing empathic responses of an unknown, random individual, and expressed themselves on the subjective (empathy ratings) and neural level (connectivity between TPJ and an AI region that correlated with trial-by-trial empathy ratings as well as the neural activity of the AI region). This indicates that the social transmission of empathy occurs in “random” social interactions and changes the neural responses to the misfortune of others, here their pain.

The finding that observing empathic responses in others changes empathic responses in the observer is important, because empathy is commonly related to an increase in prosocial behavior (37, 38). In line with these findings, the learning-related increase in empathy ratings was related to an increase in participants’ willingness to invest time to help another person. From a policy point of view, these results suggest that creating a highly empathic environment may enhance prosocial tendencies. On the flipside, our findings also show that the presence of non-empathic individuals can undermine empathy and prosocial motivation.

It has been shown before that empathy ratings of a group can shift individual empathic feelings and influence donations to a homeless shelter (4). Moreover, a recent study in a clinical setting showed that the assessment of a person’s pain by senior medical students is influenced by the opinion of other medical doctors, especially if they are unsure about the authenticity of the person’s pain responses (39). Going beyond these previous results, our study reveals a mechanism through which empathy is transmitted across individuals. We show that the extent to which people change their subjective and neural responses to the pain of others is predicted by the weight they give to the prediction-error signal generated by the discrepancy between expected and observed empathy ratings of others. Specifically, our results show that participants generate positive observational prediction errors if human demonstrators display a stronger empathic reaction than expected, and, as a result, increase their empathy ratings. In contrast, being confronted with individuals who show less empathy than expected results in negative prediction errors and a decrease in empathy ratings of the observer.

In addition, participants in the high and low empathy groups predominantly updated their values based on positive and negative observational prediction errors, respectively. Substantiating these findings, additional analyses showed stronger learning for positive compared to negative prediction errors in the high empathy group and the reverse pattern for the low empathy group (see *SI Appendix*, *Supplementary Results, Model-independent analyses on prediction errors*, for details). Previous research showed that people may perform precision weighting to discount atypical prediction errors (40, 41). In the context of these results, the asymmetric updating here may reflect the discounting of atypical prediction errors (e.g., negative prediction errors in the high and positive prediction errors in the low empathy group) to maintain the initial changes in empathy ratings (e.g., increase/decrease empathy ratings in the high/low empathy group, see *SI Appendix*, supplementary results section, simulation analyses, for details).

It is well established that observational learning parameters can predict differences in socially relevant phenomena such as the social transmission of fear (13–15), and the social modulation of risk (9) and choice preferences (7, 8). In influential theoretical models, observational learning has long been assumed to constitute a mechanism for the social transmission of empathy (5). Providing empirical evidence for this notion, we show that an observational learning model can predict the extent to which empathy is transmitted from one individual (i.e., the demonstrator) to another (i.e., the observer) and applied by the observer to third parties uninvolved in the learning process (generalization).

We find that learning from observing other’s empathic reactions does not only change participants’ empathy ratings, but also their neural responses to other’s pain. Specifically, the weight participants assigned to observational prediction errors modulated connectivity between regions associated with observational learning, such as the TPJ (12, 16, 42), and regions associated with the processing of other’s pain, such as the AI (19, 27–29, 31, 32). Taking an individual difference perspective, the more strongly a person weighted the observational prediction errors, the stronger the coupling of left TPJ–AI in the high empathy group, and the weaker the TPJ–AI coupling in the low empathy group. Apart from this, the individual differences in the magnitude of observational learning (i.e., weight parameter) also modulated the neural activations in the AI. Thus, the empathy shown by the role model modulated the way in which observational prediction error weights affected brain connectivity.

The finding of the processing of observational prediction errors in the left TPJ is in line with recent evidence linking this region to social influence on reward learning (12, 16) and prosocial decision making (43). Extending these previous results, our findings show that learning by observing high and low empathic individuals modulates the connectivity between the left TPJ and the AI as well as the vmPFC. Importantly, the AI region that was modulated by learning also significantly correlated with the trial-by-trial empathy ratings in the baseline session. Therefore, observational learning indeed changed the processing of other’s pain in the AI, i.e., a region that forms a central part of the empathy network (19, 27–29, 31, 32).

Neural responses in the vmPFC have been related to value computation in general (44), and in particular, to the computation of the value of pain (45). Given the present findings, it is possible that observing empathic responses of others changes participants’ valuation of the pain of others to justify an increase or decrease in participants own empathy ratings. Together, our neural findings uncover a neural mechanism for the social transmission of empathy that can explain the plasticity of empathic responses in different social environments.

Although we show a change in empathy ratings and neural responses to the pain of others that is closely predicted by learning parameters, alternative explanations to observational learning have to be considered. First, the observed changes in subjective and neural empathy responses may reflect mere imitation of motor responses. The results of the non-social control study argue against this alternative explanation. Although participants paid attention to, and learned to predict, the computer-generated ratings equally well as those of the human demonstrators, they did not use the learned information to update their own empathy as much as with human demonstrators. Second, participants may have changed their ratings because they were instructed to predict the ratings of the demonstrator (resulting in an “implicit instruction” effect). Our results replicate learning-related changes in empathy when participants were not instructed to predict the demonstrators’ ratings (Study 4), rendering the possibility unlikely that the observed effects mainly reflect implicit instruction demands. Third, it is possible that participants changed their empathy ratings to conform with the ratings of the demonstrator or may have shown higher empathy ratings in the high-empathy group to please the demonstrator or the experimenter. Testing these assumptions, we found no significant relationship between participants’ ratings on a well-established conformity (46) and social desirability (22) scales and their changes of empathy ratings in the observational-learning-of-empathy task. In addition, in the behavioral replication studies, participants were seated alone during the experiment, such that they were unobserved and could not interact with the experimenter. Although this setting minimized the influence of social desirability, the findings still replicated the learning-related changes in empathy ratings observed in the fMRI study. Based on this evidence, and given that the estimates from our observational learning model fitted the changes in empathy ratings and neural responses to other’s pain, observational learning is likely to contribute to the social transmission of empathy.

We report findings from four independent samples that all consisted of young female participants. This allowed us to control for unspecific gender effects (e.g., induced by gender-mixed pairings of participants and confederates), and well-established age differences in decision-making (47), prosocial tendencies (48, 49), and the processing of rewards (50). The results of a replication study in a Caucasian sample show that the observational learning of empathy is also evident in participants from a different ethnicity (Caucasian instead of Asian) that are slightly older than the samples in the current studies (see *SI Appendix*, *Supplementary Results* for details). This result suggests that our findings generalize to other groups of individuals. That said, future research should investigate observational learning of empathy in males and across the life span. Moreover, given evidence that the influence of social information decreases with increasing intelligence quotient (IQ) (51), individual differences in intelligence and cognitive abilities should be taken into account. Finally, although our results show that learning from observing the empathic reactions of a demonstrator changes the willingness of observers to invest time to help a recipient of pain, it would be important to investigate changes in actual prosocial behavior in real life.

In sum, our study shows how empathy spreads in social interactions and provides a computational and neural mechanism for the social transmission of empathy.

## Materials and Methods

### Participants.

We conducted four studies in sequential order and recruited the participants accordingly. Importantly, the four different samples were matched with regard to variables that are relevant in the context of our study (Table 1).

**Table 1.** Sample characteristics of Studies 1–4.

	Study 1	Study 2	Study 3	Study 4	ANOVA	
Variables	Mean ± SD	Mean ± SD	Mean ± SD	Mean ± SD	F-value	*P*
Age	21.1 ± 2.1	20.8 ± 2.4	20.7 ± 1.9	20.9 ± 2.2	0.285	0.836
Education	15.5 ± 1.7	15.4 ± 2.2	15.0 ± 1.7	15.6 ± 1.9	1.11	0.344
IRI	96.7 ± 10.3	97.2 ± 10.0	99.1 ± 11.9	98.2 ± 9.2	0.588	0.624
Contagion	22.6 ± 3.8	22.4 ± 3.5	23.0 ± 3.7	23.2 ± 3.6	0.604	0.613
Empathy	23.3 ± 4.5	22.2 ± 3.9	22.7 ± 4.3	22.7 ± 4.3	0.577	0.631
SDS	10.1 ± 3.3	9.1 ± 2.7	9.2 ± 3.0	8.8 ± 3.0	1.865	0.137
Conformity	53.9 ± 10.3	54.0 ± 12.7	51.5 ± 13.6	57.1 ± 12.6	1.846	0.140

Education = Previous years of education; IRI = Interpersonal Reactivity Index (measure of trait empathy); SDS = Social Desirability Scale; Contagion = Behavioral Contagion; Empathy = Empathy Index.

#### *Study 1: fMRI study*.

Fifty-five healthy Asian females (mean age ± SD = 21 ± 2.1 y) participated in the fMRI study as paid volunteers. We chose an all-female instead of a gender-mixed group of participants so that we could also use all-female confederates and avoid the complications of the gender-mixed pairing of participants and confederates. Three participants were excluded from further analyses due to excessive head movements (>3 mm) during scanning. The analyses included data from 52 participants (mean age ± SD = 21 ± 2.1 y; 26 in the high empathy group).

#### *Study 2: Non-social control study*.

Fifty-seven healthy Asian females (mean age ± SD = 20.8 ± 2.4 y) participated in the non-social control study as paid volunteers. One participant was excluded because of technical issues during the experiment. Data from 56 participants were analyzed (26 in the high empathy group). Of these, one participant did not fill in the questionnaire (see below).

#### *Study 3: Behavioral replication study*.

Fifty-six healthy Asian females (mean age ± SD = 20.8 ± 1.9 y) participated in the behavioral replication study as paid volunteers. Four participants were excluded because of technical issues during the experiment. Data from 52 participants were analyzed (25 in the high empathy group).

#### *Study 4: Behavioral replication study without prediction*.

Fifty-six healthy Asian females (mean age ± SD = 20.9 ± 2.2 y) participated in the behavioral study in which they were no longer asked to predict the demonstrators’ ratings as paid volunteers. One participant was excluded because she did not finish the experiment. Data from 55 participants were analyzed (27 in the high empathy group*).*

The experimental procedures from all studies were approved by the local Research Ethics Committee of Peking University, Beijing, China (No. 2018-01-04). Participants were recruited through flyers/online platforms at Peking University, Beijing, China. All participants had normal or corrected-to-normal vision, no history of psychological or neurological disorders, and provided written informed consent after the experimental procedure had been fully explained. Participants were reminded of their right to withdraw at any time during the study. The sample size for the studies was determined by an a priori power analysis using G*Power 3.1 (52) for a within-between interaction in a repeated-measures ANOVA design with two groups (groups: high empathy, low empathy) and two measurements (time: before learning, after learning). A total sample size of 46 participants (23 participants per group) was required for each study to detect a medium effect size of f = 0.25 at α = 0.05 (two-tailed) with a power of 90%. We recruited more than 46 participants in all studies to account for possible data loss.

### Questionnaires.

In Studies 1, 3, and 4 (i.e., the studies with human demonstrators), participants rated their impression of the demonstrator before and after the experiment (19, 21, 28, 53). In addition, participants rated the perceived empathy of the demonstrator (“How empathic do you find this person?”) from 1 (not empathic at all) to 9 (extremely empathic). In Study 1, participants also rated the perceived pain intensity of the recipient (“How much pain did the person in the video clip experience?”) from 1 (none at all) to 9 (extreme) and indicated how much time they would like to spend comforting the recipient (0 to 60 min in 1 min increments), an item that was used to measure prosocial tendencies in previous studies (54).

Participants of all studies completed the social desirability scale [SDS-17, (22)] as well as the conformity scale (46) to measure their propensity to respond in a socially desirable manner and their tendency to conform to others. We used the IRI (20) and the subscales measuring empathy and behavioral contagion from the Empathy Index (55) to measure trait empathy. There were no differences in these trait measures as well as the age and years of education across the four studies (*ps* > 0.137, Table 1). We also compared the trait measures between groups (i.e., high and low empathy group) within each study, and the results revealed no significant difference in these trait measures between the high and low empathy group for all studies (*ps* > 0.068, *SI Appendix*, Table S6).

### Preparation and Validation of the Stimulus Set.

For the purpose of this study, we recorded videos of four different females receiving painful and non-painful stimulation. These videos were further validated through an independent online study (see *SI Appendix*, *Supplementary Methods, Preparation and validation of the stimulus set* for further details).

## Experimental Design and Procedure

### Study 1: fMRI Study.

#### *Prescanning procedure*.

Before the experiment, participants briefly met two other individuals (confederates who were not known by the participant) who were trained to act as demonstrators during the observational learning task. Participants and confederates were instructed together and were told that they would be asked to indicate how they felt when watching video clips showing recipients receiving painful or non-painful stimulations on a scale from 0 (did not feel anything) to 100 (feeling extremely bad). To provide a first-hand experience of the stimulation they would observe in recipients, participants and confederates entered into a private room successively to estimate their pain threshold following a standard procedure (19, 56, 57). Details about the prescanning procedure and the pain threshold assessment are provided in *SI Appendix*, *Supplementary Methods, Prescanning procedure*.

#### *Scanning procedure*.

The fMRI scanning session consisted of a baseline session, an observational empathy learning session, and a generalization session.

In the baseline session, the participant in the scanner watched the video clips of a person receiving either painful (18 trials) or non-painful (12 trials) stimulations. Each trial started with a lightning bolt symbol (1,000 ms) indicating the pain intensity the recipient was about to receive (bright = painful; dark = non-painful). After a fixation period (500 to 1,500 ms), the video showed the hand of the recipient undergoing stimulation for 2,000 ms. Participants were then asked to report their current feelings from 0 (felt nothing at all) to 100 (felt extremely bad) within 5,000 ms.

The observational empathy learning session was adapted from an observational learning paradigm we used previously (7, 8). In each trial, an observation phase (i.e., observing the demonstrator’s empathy ratings) was followed by a self-rating phase (i.e., making empathy ratings oneself; Fig. 1). To distinguish the two phases and the different demonstrators, the beginning of each phase was marked with arrows in different colors (500 ms) pointing away (observation phase) or toward (self-rating phase) the participant. During the observation phase, the lightning bolt symbol (1,000 ms) was shown followed by the presentation of a video clip (2,000 ms). Participants were told that the demonstrator had watched this video and rated her feelings. Then, participants had 5,000 ms to predict the demonstrator’s ratings. After that, the rating of the demonstrator was presented (2,000 ms). Next, an arrow pointing to the participant indicated the start of the self-rating phase (500 ms). After the presentation of the lightning bolt (1,000 ms) and the video clip (2,000 ms), participants were asked to rate how they felt when watching the video on a scale from zero (not feeling anything) to hundred (feeling extremely bad) (5,000 ms). The videos used in the observation and the self-rating phase showed the same recipient receiving the same type of stimulation (i.e., either depicting painful or non-painful stimulation).

The observational empathy learning session consisted of four blocks, with 12 trials in each block, resulting in 48 trials in total (36 trials of painful and 12 trials of non-painful videos). To prevent habituation, participants saw the video clips of two different recipients (one recipient for two blocks) in the observational empathy learning session.

Unbeknownst to the participants, the ratings of all demonstrators were generated by a pre-defined algorithm, based on the participant empathy ratings in the baseline session. In the high empathy group, the observed ratings for pain videos were drawn from a normal distribution in which the mean equaled the participant mean in the baseline session plus three SD (SD = 5). In the low empathy group, they were drawn from a normal distribution in which the mean equaled the participant mean in the baseline session minus three SD (SD = 5). As a result, in the high empathy group, participants observed empathy ratings that were consistently higher, and in the low empathy group, they observed ratings that were consistently lower than their baseline ratings for the painful videos. The observed ratings for non-painful videos were sampled from a normal distribution in which the mean of the distribution was the individual mean in the baseline session (SD = 5).

The generalization session was identical to the baseline session, except that the participants provided emotion ratings when observing a new recipient, i.e., video clips that were not part of the baseline or the observational empathy learning session. The participant and confederates were informed that they would not meet after the study and had separate visual displays to keep empathy ratings anonymous.

### Study 2: Non-Social Control Study.

The task of the control study was identical (i.e., instructions, number of sessions, number of blocks, and number of trials) to the task of the fMRI study described above, except that participants were told that they observed ratings generated by two computers.

### Study 3: Behavioral Replication Study.

To test the robustness of the learning effects observed in the fMRI study, we conducted a behavioral study on an independent sample. The experimental procedure was identical to the procedure of the fMRI study described above, except that the demonstrators were represented by real participants instead of confederates. Care was taken to ensure that the participants had neither met nor known each other before the study. To further minimize a potential effect of reputation concerns on empathy ratings, participants were seated alone in the laboratory, i.e., the experimenter was not present and did not interact with the participants during the experiment. Importantly, the ratings of the demonstrators in the observational-learning-of-empathy session were also generated with the pre-defined algorithm described above.

### Study 4: Behavioral Replication Study without Prediction.

The experimental procedure and the task were identical to Studies 1 and 3, except that participants were no longer asked to predict the ratings of the demonstrator. The prediction screen of the observation phase (Fig. 1 *C*, *Upper* panel) that was shown in all other studies was deleted in Study 4. This measure was taken to rule out the possibility that observed changes in empathy ratings reflect demand effects induced by the instruction to predict the ratings of the demonstrator.

### MRI Acquisition.

We acquired functional and anatomical images with a Siemens Trio 3.0 T MR scanner using a 12-channel phase-array head coil at the Center for MRI Research, Peking University. Multiband functional images were acquired with T2-weighted, gradient-echo, echo-planar imaging sequences sensitive to BOLD contrast (matrix = 112 × 112, 62 slices, 2 × 2 × 2 mm^3^ voxel size, interslice gap = 0.3 mm, repetition time (TR) = 2,000 ms, echo time (TE) = 30 ms, field of view (FOV) = 22.4 × 22.4 cm, flip angle (FA) = 90°, interleaved slice acquisition, multiband acceleration factor = 2). A high-resolution anatomical T1-weighted image was acquired for each participant (256 × 256 mm matrix, 192 slices, 1 × 1 × 1.00 mm^3^ voxel size; TR = 2,530 ms, TE = 2.98 ms, inversion time (TI) = 1,100 ms, FOV = 25.6 × 25.6 cm, FA = 7°). Padded clamps were used to minimize head motion and earplugs attenuated scanner noise.

### Data Analyses.

#### *Regression analyses*.

We performed linear mixed models (LMM, “lme4”) in R v.4.1.1 (R Development Core Team, 2012) for the behavioral analyses on empathy ratings and prediction ratings as the dependent variables to investigate observational learning. In particular, we conducted LMMs with empathy group (high empathy, low empathy), time and empathy group × time as predictors, and the empathy ratings or prediction ratings as the dependent variable to identify the change of empathy or prediction ratings across time. In addition, we performed LMMs to compare observational learning effects as captured by computational models between studies. Specifically, study (fMRI, non-social control/ behavioral replication/behavioral replication without prediction), empathy group (high empathy, low empathy), and trial-wise observational prediction errors (obtained in the RL model) as well as their interactions were included as a fixed effect to predict the trial-wise changes of empathy ratings. We also used by-participant intercepts for all LMMs (see *SI Appendix*, *Supplementary Methods, Regression analyses* for details).

#### *Computational modeling*.

To investigate the mechanisms underlying changes in empathy on a trial-by-trial basis in the observational learning session, we employed a computational modeling approach (21, 25, 26). Specifically, first we modeled the predictions participants made regarding the ratings of the demonstrators using a standard Rescorla–Wagner (24) RL algorithm in the observation phase. Next, we modeled participants’ empathy ratings as a linear combination of the time-discounted sum of previous observational prediction errors (as originating from the RL model) and participants’ baseline ratings ($Empathy\left(t0\right)$), which were defined as the individuals’ mean ratings toward painful videos in the baseline session when no social influence was implemented.

We optimized model parameters by minimizing the negative logarithm of the posterior probability (LPP) and computed the Laplace approximations to the model evidence (LAME) as criteria for model comparison, which measure the ability of each model to explain the experimental data, by trading-off their goodness-of-fit and complexity (58, 59). Further information about model specifications, model fitting procedure, parameter estimation, and model comparison are provided in *SI Appendix*, *Supplementary Methods, Computational modeling*.

### MRI Image Analyses.

#### *Preprocessing, first-level, and second level analysis*.

fMRI data were analyzed using SPM12 (https://www.fil.ion.ucl.ac.uk/spm/) using a standard preprocessing pipeline (see *SI Appendix*, *Supplementary Methods, fMRI analyses*, for further details). We assessed the regions encoding the trial-by-trial empathy ratings in the baseline and in the generalization session, the regions encoding the trial-by-trial observational prediction errors in the observational learning session, and compared high against low empathy groups (see *SI Appendix*, *Supplementary Methods, fMRI analyses*, for further details on the first level and second level analysis).

#### *Psychophysiological interaction (PPI) analyses*.

To examine how neural activity related to observational prediction errors influences neural responses in the self-rating phase and lead to the differential responses between high and low empathy groups, we performed PPI analyses (60, 61). In particular, we assessed whether brain regions that were related to observational prediction errors (Fig. 4*D*) changed their functional connectivities with other regions depending on the individual size of the *W1* parameter, i.e., the parameter associated with the change in empathy across participants in the behavioral analyses (Fig. 3 *G* and *H*, see *SI Appendix*, *Supplementary Methods, fMRI analyses*, for further details on the PPI analyses). Imaging results were determined in whole-brain analyses, using a combined voxel-level threshold of *P*_uncorrected_ < 0.001 and an FWE-corrected cluster-level threshold of *P* < 0.05.

## Supplementary Material

Appendix 01 (PDF)

## Data Availability

The data and codes support the findings of the current study are available at https://osf.io/n49y3/?view_​only=60dd2d738b2646d6ada135aa1913f7dd (62).
