# Supplementary material for: The social transmission of empathy relies on observational reinforcement learning
Source: Proc Natl Acad Sci U S A. 2024 Feb 21;121(9):e2313073121. doi: 10.1073/pnas.2313073121 (PMC10907261; doi:10.1073/pnas.2313073121)
Supplement: Supplementary file 1 — Appendix 01 (PDF) [file pnas.2313073121.sapp.pdf]

PNAS

Supporting Information for

The social transmission of empathy relies on observational reinforcement learning

Yuqing Zhou*, Shihui Han, Pyungwon Kang, Philippe N. Tobler & Grit Hein*

Address correspondence to:
Prof. Dr. Grit Hein,
Email: Hein_G@ukw.de

Dr. Yuqing Zhou,
Email: zhouyq@psych.ac.cn

This PDF file includes:

Supplementary Methods
Preparation and validation of the stimulus set
Prescanning procedure
Regression analyses
Computational modeling
fMRI analyses

Supplementary Results
Behavioral replication study without prediction
Behavioral replication with a sample from a different ethnicity
Model independent analyses on prediction error
Simulation analyses

Supplementary Figure Fig S1~S6
Supplementary Table S1~S6

SI references

Supplementary Methods

Preparation and validation of the stimulus set

In each video clip, two pain electrodes were visibly attached to the recipient's right hand. The recipient reacted to the shocks by twitching her hand and arm when receiving a painful electrical stimulation and acted calmly when receiving a non-painful electrical stimulation. For each recipient, we recorded at least 10 video clips showing painful stimulation and 4 video clips showing non-painful stimulation with a duration of 2 s each. We then selected 25 out of the 40 video clips showing painful stimulations for further stimulus validation.

To validate the video clips, we conducted an online study with 37 female participants (mean age ± SD = 21.9 ± 4.4 years). The rating task was completed electronically via a Qualtrics link (https://www.qualtrics.com/). Participants were instructed to watch the 25 video clips and to rate the pain intensity felt by the recipient ("How painful do you think the model feels?") on a 7-point Likert scale (1 = not painful at all, 7 = extremely painful). The order of the presentation of the video clips was randomized. Based on these ratings, we selected four video clips showing painful stimulation for each recipient (16 video clips in total). We then averaged the pain intensity ratings for each recipient and conducted further statistical tests. The mean pain intensity ratings were comparable across recipients ($F(3,34) = 0.473$, $p = 0.703$, η^2_p, = 0.040, **Figure S1**).

Prescanning procedure

Before the experiment, participants briefly met two other individuals (confederates who were not known by the participant) who were trained to act as demonstrators during the observational learning task. Participants and confederates were instructed together. They were told that the current study was part of a project on pain perceptions and that they would be randomly assigned to one of two groups; a 'recipient' group that would receive painful or non-painful electrical stimulations, or an 'observer' group that would watch the stimulation of the recipients and rate their feelings. The participants and the two confederates were ostensibly assigned to the 'observer' group.

Next, the individual pain thresholds of the participants and confederates were determined by a standard procedure (1–3) to provide a first-hand experience of the stimulation they would observe in recipients. To do so, participants and confederates entered a private room successively in which another experimenter performed the pain threshold assessment. More specifically, two pain electrodes were attached to the back of the left or right hand. Using a Digitimer DS7 electrical stimulator, a low-voltage electric shock (0.5 mA) was delivered and increased in increments of 0.5 mA. Participants and confederates were asked to rate the intensity of the respective electrical stimulation from 0 (not painful at all) to 10 (extremely painful). Participants and confederates were informed that the recipients would receive pain stimulation with the intensity they rated as "8" and non-painful stimulation with the intensity they rated as "1" in the pain thresholding procedure.

After measuring individual pain thresholds, the experimenter introduced the empathy rating scale. Participants and confederates were told that they would be asked

to indicate how they felt when watching a video clip of a recipient on a scale from 0 (did not feel anything) to 100 (feeling extremely bad). Next, the participants received instructions for the observational empathy learning task in the preparation room while the two confederates were seated outside. They were then instructed that apart from reporting their feelings when watching the video clips, their task would be to predict the ratings of the demonstrators (i.e., the two confederates) as accurately as possible. To help with their predictions, the participants would see the rating of the demonstrator in real time after their prediction. We made clear to the participants that their own ratings were personal and could not be observed by others.

Regression analyses

We performed linear mixed models (LMM, ‘lme4’) in R v.4.1.1 (R Development Core Team, 2012) for the behavioral analyses on empathy ratings and prediction ratings as the dependent variables to investigate observational learning. In particular, we conducted LMMs with empathy group (high empathy, low empathy), time and empathy group × time as predictors, and the empathy ratings or prediction ratings as the dependent variable. The time variable corresponds to the trial number (i.e., 1-36 trials) during the observational empathy learning session or the session number of the whole experiment (baseline, observational empathy learning (1-4) and generalization, coded as 0-5 respectively). We predicted significant empathy group × time interactions for both the empathy ratings and the prediction ratings. Specifically, we hypothesized that participants’ prediction and empathy ratings would diverge between high and low empathy groups over the course of learning. We used participants as random intercepts.

In addition, we performed LMMs to compare observational learning effects as captured by computational models between studies. Specifically, experiment (fMRI, non-social control/ behavioral replication/behavioral replication without prediction), empathy group (high empathy, low empathy), and trial-wise observational prediction errors (obtained in the reinforcement learning model) as well as their interactions were included as a fixed effect to predict the trial-wise changes of empathy ratings. We also used by-participant intercepts for all LMMs.

Likelihood ratio tests were applied to assess the significance of the fixed effects. The resulting χ^2 values indicate how much more likely the data are under the assumption of a more complex model (i.e., a model including a particular parameter) than under the assumption of a simpler model (i.e., a model not including this parameter).

Computational modeling

To investigate the mechanisms underlying changes in empathy on a trial-by-trial basis in the observational learning session, we employed a computational modeling approach (4–6). Specifically, first we modeled the predictions participants made regarding the ratings of the demonstrators using a standard Rescorla-Wagner (7) reinforcement learning (RL) algorithm in the observation phase. Next, we modelled participants’ empathy ratings as a linear combination of the time-discounted sum of previous observational prediction errors (as originating from the RL model) and participants’

baseline ratings ($Empathy(t0)$), which were defined as the individuals' mean ratings towards painful videos in the baseline session when no social influence was implemented. The results were based on the original (raw) ratings. Using normalized ratings revealed similar results. First, we modeled the predictions participants made regarding the ratings of the demonstrators using a standard Rescorla-Wagner (7) reinforcement learning (RL) algorithm in the observation phase. The RL model assumes that participants changed their predictions when the demonstrator ratings differed from the ratings expected by the participants.

$$V(t+1) = V(t) + \alpha \times \delta_i \quad [1]$$

$$\delta_i(t) = R(t) - V_i(t) \quad [2]$$

Thus, on each trial *t*, the (future) predictions $V(t+1)$ of demonstrator ratings are a function of current predictions $V(t)$ and the prediction error δ (Equation 1), which corresponds to the difference between the actual demonstrator rating *R(t)* at trial *t* and the current prediction $V(t)$ (Equation 2). In our study, the demonstrator's rating can be higher or lower than expected. Observing higher ratings than expected generates a positive prediction error, while observing lower ratings than expected generates a negative prediction error. The learning rate α ($0 \leqslant \alpha \leqslant 1$) controls the extent to which the current predictions of demonstrators' ratings are updated by new information.

Next, we formally modelled the participants' empathy ratings in the self-rating phase as a linear function of prediction errors elicited by demonstrator's empathy ratings in the preceding observation phase. In all models, we assumed that participants' ratings are a linear combination of the time-discounted sum of previous observational prediction errors (as originating from the RL model, Equations 1-2) and participants' baseline ratings ($Empathy(t0)$), which were defined as the individuals' mean ratings towards painful videos in the baseline session when no social influence was implemented.

We considered models which separated the first and second half of the observational learning session as these two halves used different recipients of pain stimulation in the videos. Moreover, we found that empathy group (high, low) and session half (first, second) interacted for observational prediction errors ($\chi^2(1) = 19.82$, $p < 0.001$). Specifically, in the first half, the observational prediction errors were mostly positive for the high empathy group and mostly negative for the low empathy group, resulting in a group difference ($\chi^2(1) = 33.36, p < 0.001$). In contrast, in the second half, the observational prediction errors were close to zero for both groups, resulting in no difference between groups ($\chi^2(1) = 0.07$, $p = 0.79$). We also considered models with common and separate weighting of positive and negative prediction errors:

$$Empathy(t) = Empathy(t0) + W \sum_{j=1}^{t} \gamma^{t-j} \delta_j \quad [3]$$

$$Empathy(t) = Empathy(t0) + W_{pos} \sum_{j=1}^{t} \gamma^{t-j} \delta pos_j + W_{neg} \sum_{j=1}^{t} \gamma^{t-j} \delta neg_j \quad [4]$$

$$Empathy(t) = \begin{cases} Empathy(t0) + W1_{pos} \sum_{j=1}^{t} \gamma^{t-j}\, \delta pos_j + W1_{neg} \sum_{j=1}^{t} \gamma^{t-j}\, \delta neg_j, t < 25 \\ Empathy(t0) + W2_{pos} \sum_{j=1}^{t} \gamma^{t-j}\, \delta pos_j + W2_{neg} \sum_{j=1}^{t} \gamma^{t-j}\, \delta neg_j, t \geq 25 \end{cases} \quad [5]$$

$$Empathy(t) = Empathy(t0) + k \times R(t) \quad [6]$$

The winning model 3 (Equation 5) considered the empathy rating in the first and second half of the observational learning session separately, separated the prediction errors by sign, and added them up separately. This model included the parameters *W1* and *W2,* which capture the magnitude (weight) of the influence of observational prediction errors on changes in participants' empathy ratings in the first and second half of the observational learning session. The *W* parameter ranges from -1 to +1 because one represents the maximum of the empathy ratings after the transformation (i.e., divided by 100). A larger *W* corresponds to a stronger influence of observational prediction errors on participants' empathy ratings. The discount parameter γ ($0 \leqslant \gamma \leqslant 1$), captures an exponential decay of the influence of previous observational prediction errors over time, such that the more recent observational prediction errors have a greater impact on participants' empathy ratings than the earlier observational prediction errors. If γ is close to one, all preceding observational prediction errors receive the same weight, and if it is close to zero, only the last observational prediction error leads to subsequent changes in participants' empathy ratings.

We also tested less complex models in which positive and negative prediction errors were not modelled separately (Equation 3, Model 1) or the empathy ratings were not fitted separately for the first and second half of the observational learning session (Equation 4, Model 2). Moreover, we tested an imitation model in which participants were allowed to differ in the extent to which they copied the demonstrators' ratings (Equation 6, Model 4). In this model, *k* represents the imitation parameter and *R(t)* is the actual demonstrator rating at trial *t*. We fitted all computational models to participants' ratings of the painful videos in both high and low empathy groups.

Parameter estimation

We optimized model parameters by minimizing the negative logarithm of the posterior probability (LPP) over the free parameters using MATLAB's fmincon function, initialized at multiple starting points of the parameter space.

$$\text{LPP} = -\log\big(P(\theta_M|D,M)\big) \propto -\log(P(D|M,\theta_M)) - \log(P(\theta_M|M))$$

Here, $P(D|M,\theta_M)$ is the likelihood of the data given the considered model M and parameter values θ_M, and $P(\theta_M|M)$ is the prior probability of the parameters. Following previous research (8), the prior probability distributions for the learning rate were defined as beta distributions (beta pdf(α,1.1,1.1)). For the weight parameters and forgetting parameters, the prior distributions were unknown and assumed to be uniform,

such that every value in the parameter range had equal probability. Formally, this is equivalent to maximum likelihood estimation (9).

Model comparison

We computed the Laplace approximations to the model evidence (LAME) as criteria for model comparison, which measure the ability of each model to explain the experimental data, by trading-off their goodness-of-fit and complexity (8, 10).

$$LAME = \log(P(D|M, \theta_M)) + \log(P(\theta_M|M)) + \frac{df}{2}\log 2\pi - \frac{1}{2}\log|H|$$

Where df is the number of model parameters, and |H| is the determinant of the Hessian.

The individual model comparison criteria (LAME) were then fed to the mbb-vb-toolbox (https://code.google.com/p/mbb-vb-toolbox/). For each model within a set of models, we estimated the exceedance probability (denoted XP), given the data gathered from all subjects. XP quantified the belief that the model was more likely than all the other models in the model space. An XP > 95% for one model within a set is typically considered as significant evidence in favor of this model being the most likely.

fMRI analyses

Preprocessing

Functional images were slice-time corrected, realigned, and coregistered to the anatomical image of the participant. The anatomical image was processed using a unified segmentation procedure combining segmentation, bias correction, and spatial normalization to the MNI template (11), the same normalization parameters were then used to normalize the EPI images. Lastly, the functional images were spatially smoothed using an isotropic 6 mm full-width at a half-maximum (FWHM) Gaussian kernel.

First-level analysis

We first sought to identify neural regions that tracked trial-by-trial empathy ratings. To do so, we interrogated event-related general linear models (GLMs) in the baseline session. We included the onsets and durations of (1) the lightning bolt indicating the level of pain intensity; (2) the videos of recipients undergoing electrical stimulations, parametrically modulated by the trial-by-trial empathy ratings of participants; and (3) participant ratings. These regressors were convolved with the canonical hemodynamic response function and its time derivatives. The model also contained six (three translation and three rotation) regressors to account for motion.

To examine neural activity correlating with observational prediction errors, we investigated GLMs for the observational empathy learning session. We included the onsets and durations of: (1) the cues indicating the beginning of the observation phase or self-rating phase; (2) the electric bolt indicating the level of pain intensity (modelling painful and non-painful stimulations separately); (3) the videos of recipients receiving electrical stimulations (modelling separately for the painful and non-painful stimulation videos in the observation phase and self-rating phase); (4) the prediction of the demonstrator rating (modelled separately for the painful and non-painful stimulations);

(5) the ratings of the demonstrator (modelled separately for the painful and non-painful stimulations), parametrically modulated by observational prediction errors derived from the reinforcement learning model (see computational model for details); and (6) participant ratings. These regressors were again convolved with the canonical hemodynamic response function and its time derivatives, and the model contained six (three translation and three rotation) regressors to account for motion. The results are based on the original (raw) ratings. Using normalized ratings revealed similar results.

Second-level analysis

First, we assessed the regions tracking the trial-by-trial empathy ratings in the baseline session. We brought the first-level contrast images created by the parametric modulator of empathy ratings to the second level and tested against zero in a one-sample t-test.

Next, we investigated the regions encoding observational prediction errors. First, we investigated the high and low empathy groups separately and identified regions encoding observational prediction errors (i.e., by setting the prediction error regressor to "1") or inverse observational prediction errors (i.e., by setting the prediction error regressor to "-1") in one-sample t-tests at the second level.

We then collapsed all contrast images created by the observational prediction error parametric modulator from the first level and compared them between high and low empathy group at the second level. Imaging results were obtained in whole-brain analyses, using a combined voxel-level threshold of $P_{\text{uncorrected}} < 0.001$ and a family-wise error (FWE) corrected cluster-level threshold of $P < 0.05$.

Psychophysiological interaction (PPI) analyses

To examine how neural activity related to observational prediction errors influences neural responses in the self-rating phase and lead to the differential responses between high and low empathy groups, we performed psychophysiological interaction (PPI) analyses (12, 13). We used the generalized PPI (gPPI) toolbox (https://www.nitrc.org/projects/gppi), which has the benefit of accommodating multiple task conditions in the same connectivity model (14). Given that multiple regions were associated with the differential encoding of observational learning prediction errors between groups (i.e., **Table S3C**), we first conducted a multi-region PPI analysis (15) to identify brain regions that changed their functional connectivities with other regions depending on the individual size of the *W1* parameter, i.e., the parameter associated with the change in empathy across participants in the behavioral analyses, **Figure 3G** and **3H**). To do so, we defined regions of interest (ROIs) using the full set of activated clusters related to the differential processing of observational prediction errors between groups (**Table S3C**). Next, we used each of these ROIs as a seed and obtained the respective connectivity strengths with other regions across the whole brain (264 regions based on an established template (16) when participants watched others in pain in the self-rating phase (vs. the implicit baseline). Finally, we correlated the connectivity strength with the *W1* parameter. To prevent arbitrariness in the definition of the seed region, we defined it with different thresholds, ranging from 0.001 to 0.05, which led to similar conclusions (see (15) for a similar approach).

The multi-region PPI analysis revealed that the connectivity between the left TPJ and the rest of the brain showed the strongest modulation by the *W1* parameter. As such, we focused on the left TPJ in a follow-up PPI analysis. We extracted the time series of the left TPJ (the region tracking the observational prediction error) as the physiological regressor. Psychological regressors were then convolved onset regressors and parametric modulators. Psychophysiological interaction (PPI) terms were created by multiplying the time series from the psychological regressors with the physiological variable. All of the above were performed for each participant separately, and individual gPPI models were created by including the physiological variables, the psychological regressors, and the PPI terms (14).

The physiological, psychological, and psychophysiological interaction regressors as well as six motion parameters were then entered into the GLM. We first used this GLM to determine regions in which connectivity strength with the left TPJ was modulated by watching painful videos in the self-rating phase (vs. the implicit baseline) or the observation phase (vs. implicit baseline for a control analysis) in the first-level analyses. Thus, we put a weight of 1 on the PPI regressor in which the corresponding psychological regressor was the onset time when participants watched painful videos in the self-rating phase or in the observation phase, and a weight of 0 on all other regressors at the first level. Next, we determined regions whereby connectivity strength to the left TPJ was modulated by the weight given to observational prediction errors. To do so, we conducted second-level covariate analyses in which the contrast image obtained for the first-level gPPI analysis was entered into a full-factorial design, with the individual *W1* parameters in the first session (see computational models for details) as the covariates. We entered the $W1_{pos}$ for the high empathy group and $W1_{neg}$ for the low empathy group. We tested the functional connectivity that was differentially associated with the *W*1 parameter in the high and low empathy group. Imaging results were determined in whole-brain analyses, using a combined voxel-level threshold of $P_{\text{uncorrected}} < 0.001$ and an FWE-corrected cluster-level threshold of $P < 0.05$.

The PPI analysis revealed that the individual *W1* parameters modulated the connectivity between the left TPJ and the left AI in the self-rating phase. In additional analyses, we aimed to specify the function of the AI that was identified in the PPI analysis. Using the identified AI region (**Figure 5**, upper panel) as a mask for small-volume-correction (FWE-SVC < 0.05), first we regressed the individual *W1* parameters against the neural activity to the painful videos in the self-rating phase using a second-level regression. Second, we compared the neural activity tracked by the trial-by-trial empathy ratings between baseline session and generalization session between high and low empathy groups

Using MarsBaR (http://marsbar.sourceforge.net), we extracted beta values of identified clusters to visualize the correlations of the left TPJ with the left AI, and with vmPFC, and the weight parameters for high and low empathy groups respectively. Specifically, we plotted the connectivity strength for the left AI and vmPFC identified by the PPI analysis (**Figure 5**). We also extracted the activation of the left AI (**Figure 5**) in the baseline session to reveal the functional role of the AI.

Supplementary Results

Behavioral replication study without predictions

First, we tested the participants' own empathy ratings in the self-rating phase of the observational learning session, as well as across the whole experiment. The LMM with group (high empathy, low empathy), trial number and group × trial number as predictors, and participants' empathy ratings in the observational learning session as the dependent variable revealed a significant group × trial number interaction ($\chi2(1) = 20.38$, $p < 0.001$). A direct comparison of the changes of empathy ratings between studies revealed these changes were comparable to the effects observed in Studies 1 and 3 in which participants had to predict the demonstrator's ratings ($\chi2(2) = 0.52$, $p = 0.77$). Similarly, an LMM with group (high empathy, low empathy), session (baseline session, observational empathy learning session 1-4 and generalization session, coded as 0-5 respectively) and group × session as predictors, and participants' empathy ratings in each session as the dependent variable revealed a significant group × session interaction ($\chi2(1) = 74.65$, $p < 0.001$). Separate analyses then showed a significant increase in empathy ratings across sessions in the high empathy group ($\chi2(1) = 14.04$, $p < 0.001$), and a significant decrease in empathy ratings across sessions in the low empathy group ($\chi2(1) = 68.20$, $p < 0.001$). In summary, participants' own empathy ratings in the behavioral study without showing the prediction screen resembled those of the fMRI study as well as the behavioral replication study.

We then modeled the empathy ratings using the winning model described above (i.e., Equation 5) and the model fitted the data adequately for both the high and low empathy groups (r^2 (mean ± SD) = 0.24 ± 0.20 and 0.23 ± 0.18, **Figure S3**). Next, we extracted the trial-wise observational prediction errors and then fitted an LMM to directly test the association between prediction errors and changes in empathy ratings. The results revealed that trial-wise observational prediction errors positively predicted the trial-wise changes of empathy ratings ($\chi2(1) = 14.09$, $p < 0.001$), and similarly well in the high and the low empathy group ($\chi2(1) = 0.74$, $p = 0.39$). To compare studies more thoroughly, we also interrogated an additional LMM with study (behavioral study without predictions, behavioral replication, fMRI), empathy group (high empathy, low empathy), and trial-wise observational prediction errors, as well as their interaction to predict the trial-wise changes of empathy ratings. The analysis showed that the study ×prediction errors interaction effect was not significant ($\chi2(2) = 0.59$, $p = 0.75$, **Figure 8C**, see **Table S4** for full statistical results), suggesting that participants' empathy ratings were similarly influenced by the observational prediction errors in the fMRI study, the behavioral replication study, and the behavioral study without predictions.

Behavioral replication with a sample from a different ethnicity

Participants

34 healthy Caucasian females (mean age ± SD = 24.4 ± 4.2 years) participated in the study. The study was conducted at the University of Würzburg, Germany and approved by the local ethics committee (268/18).

Experimental procedure

The experimental procedure and trial structure were identical to Studies 1 and 3 (see Methods; **Figure 1C**).

Results

We first analyzed the predictions from the observational learning phase. To this end, we conducted an LMM with group (high empathy, low empathy), trial number and group × trial number as predictors, and participants' predictions of ratings as the dependent variable. The results revealed a significant group × trial number interaction ($\chi 2(1) = 21.0$, $p < 0.001$, **Figure S4A**), indicating that participants expected increasing empathy ratings of the demonstrators in the high ($\chi 2(1) = 3.97$, $p = 0.046$) and decreasing empathy ratings in the low empathy ($\chi 2(1) = 16.31$, $p < 0.001$) group also in this independent sample.

We then tested the participants' own empathy ratings in the self-rating phase of the observational learning session. The LMM with group (high empathy, low empathy), trial number and group × trial number as predictors, and participants' empathy ratings as the dependent variable revealed a significant group × trial number interaction ($\chi 2(1) = 46.47$, $p < 0.001$), similar to the results of the Asian participants. We also analyzed the empathy ratings of our participants over the whole experiment (i.e., from the baseline session to the generalization session). To this end, we conducted an LMM with group (high empathy, low empathy), session (baseline session, observational empathy learning session 1-4 and generalization session, coded as 0-5 respectively) and group × session as predictors, and participants' empathy ratings as the dependent variable. Similar to the results of Studies 1, 3 and 4, we also found a significant group × session interaction ($\chi 2(1) = 48.99$, $p < 0.001$), with an increase in ratings across sessions in the high empathy group ($\chi 2(1) = 14.31$, $p = 0.013$) and a decrease in ratings across sessions in the low empathy group ($\chi 2(1) = 60.38$, $p < 0.001$).

To test if these changes in empathy ratings were associated with observational learning, we fitted the participants' predictions using a Rescorla-Wagner reinforcement-learning model, and then extracted the trial-wise observational prediction errors and associated them with trial-wise changes in empathy ratings in an LMM. The results revealed that trial-wise observational prediction errors positively predicted the trial-wise changes of empathy ratings ($\chi 2(1) = 9.50$, $p = 0.002$), and similarly well in the high and the low empathy group ($\chi 2(1) = 0.03$, $p = 0.85$). To compare studies more thoroughly, we also interrogated an additional LMM with study (behavioral replication with a sample from a different ethnicity, behavioral replication, fMRI), empathy group (high empathy, low empathy), and trial-wise observational prediction errors, as well as their interaction to predict the trial-wise changes of empathy ratings. The analysis showed that the study × prediction errors interaction effect was not significant ($\chi 2(2) = 2.17$, $p = 0.34$, **Figure S4B**, **Table S4**), compatible with the notion that participants' empathy ratings were similarly influenced by the observational prediction errors in the Asian sample and the Caucasian sample.

Model independent analyses on prediction error

The asymmetric weighting of positive and negative prediction errors between the high and the low empathy group suggests differential updating of empathy ratings based on the valence of prediction errors in both groups. We thus checked the change of empathy ratings following each type of prediction error in each group (the change of empathy ratings in response to negative prediction errors was reversed to make them comparable to the change of empathy ratings in response to positive prediction errors). The results of a regression analysis with group (high/low empathy group) and valence of prediction error (positive/negative) as regressors and changes in empathy ratings as dependent variable revealed a significant group × valence interaction effect ($\chi2(1) = 12.01$, $p < 0.001$). Participants in the high empathy group, compared to the low empathy group, showed more updating after positive prediction errors compared to negative prediction errors (**Figure S5A**, **Table S5**), indicating stronger learning from typical than atypical prediction errors. To further control the influence of the magnitude of prediction errors on the update of empathy ratings, we computed model-independent learning rates by dividing the change of empathy ratings by the absolute prediction errors (i.e., LR = delta empathy rating/absolute prediction errors). A regression analysis with model-independent learning rates as dependent variable and group (high/low empathy group) and valence of prediction error (positive/negative) as regressors, showed a significant group (high empathy/low empathy) × valence interaction ($\chi2(1) = 5.39$, $p = 0.02$, **Figure S5B**, **Table S5**), indicating higher learning rates for positive prediction errors compared to negative prediction errors in the high empathy group compared to the low empathy group.

Simulation analyses

We also ran simulation analyses to examine the asymmetric updating. Specifically, using the behavioral data and the averaged model parameters from our main study (Study 1), we simulated learning with an equal weight on positive and negative prediction errors in both groups, i.e., without putting a lower weight on atypical prediction errors (negative prediction errors in the high and positive prediction errors in the low empathy group). Learning with equal weights on positive and negative prediction errors would result in a decline of empathy ratings in the high empathy group and an increase in the low empathy group (**Figure S6**, black line), which is inconsistent with the observed findings (grey line). In contrast, the results of a simulation with lower weights on the negative prediction errors in the high empathy group and lower weights on the positive prediction errors in the low empathy group (red line) returned an increase of empathy ratings in the high, and a decrease of empathy ratings in the low empathy group, in line with the observed results (**Figure S6**). These results suggest that the observed learning-related change rely on a stronger weight on typical compared to atypical prediction errors to maintain the initial changes of empathy ratings.

Supplementary Figures

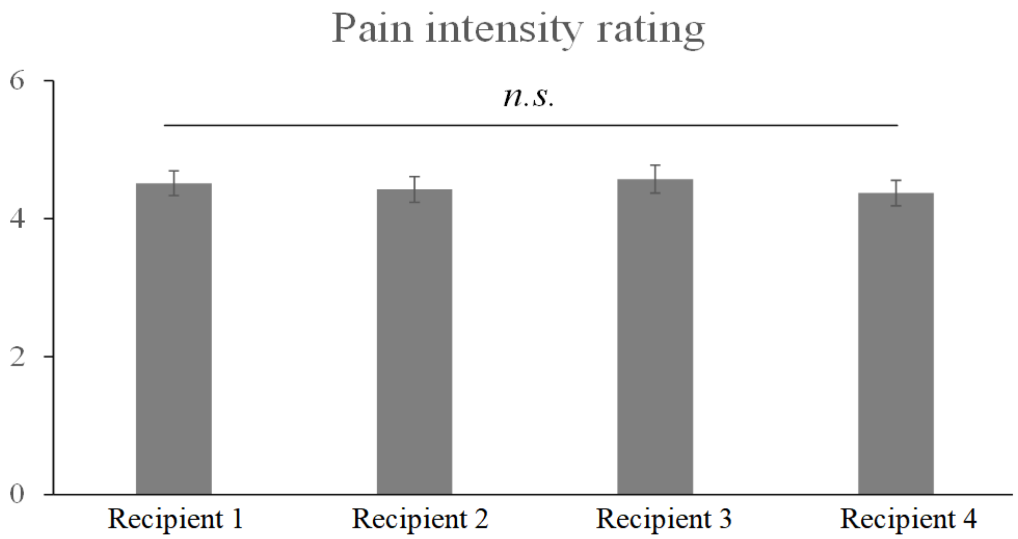

Figure S1. **Rating scores from an independent group of female participants (N = 37).** The pain intensity ratings were matched between recipients.

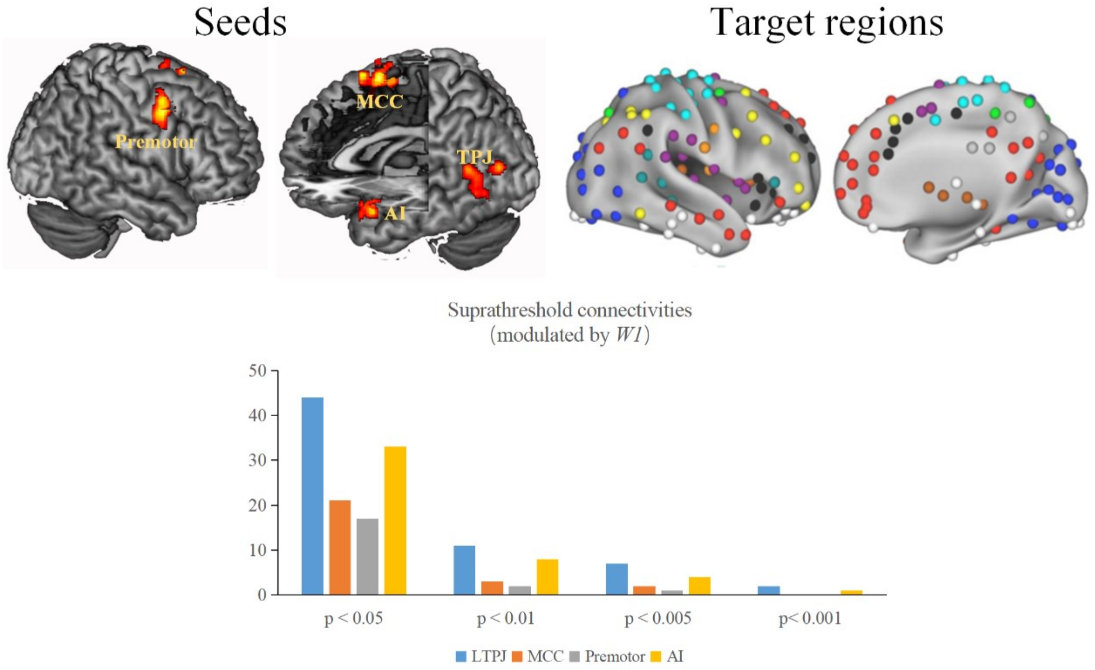

Figure S2. Multi-region PPI analysis. The upper panel shows the seeds for the multi-region PPI analysis and the target regions. The lower panel shows the number of connectivities modulated by the strength of observational learning (i.e., *W1* parameter), collapsed over high and low empathy groups.

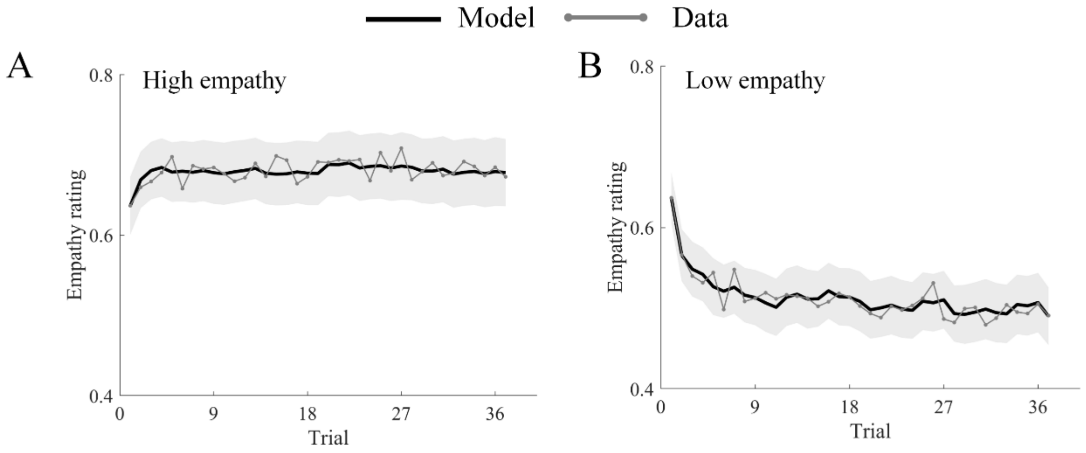

Figure S3 (A and B) Trial-by-trial empathy ratings (light grey) and corresponding model estimates (dark grey, shaded area represents the ±1 standard error) for the high and the low empathy groups in Study 4 (behavioral replication study without prediction).

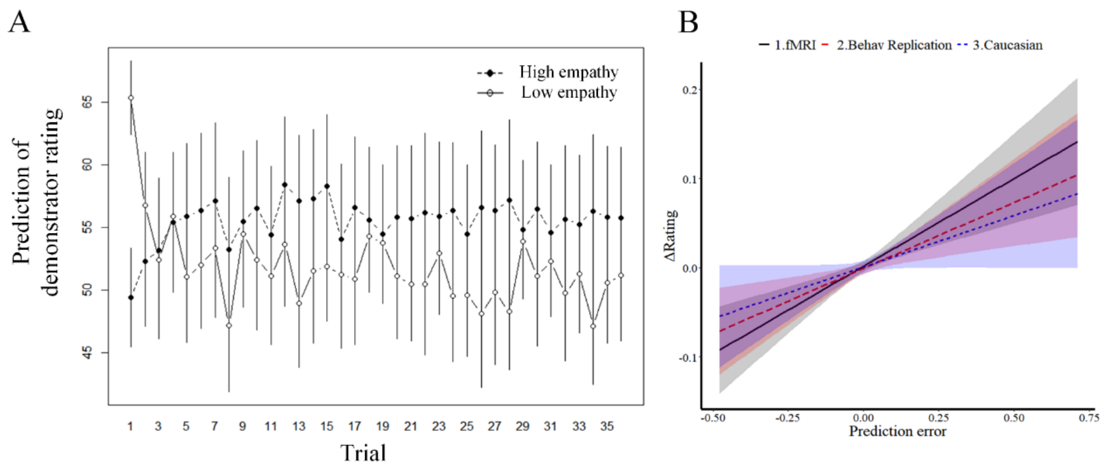

Figure S4. Results of the behavioral study conducted with participants from a different ethnicity (Caucasian). (A) Trial-by trial prediction ratings. The results showed differential effects in the high and low empathy groups. (B) Effect of prediction error on changes in empathy ratings in the fMRI study (black), the behavioral replication study (red) and the behavioral replication study with a sample from a different ethnicity (blue). The interaction between study × prediction error was not significant, indicating comparable observational learning of empathy in the three studies.

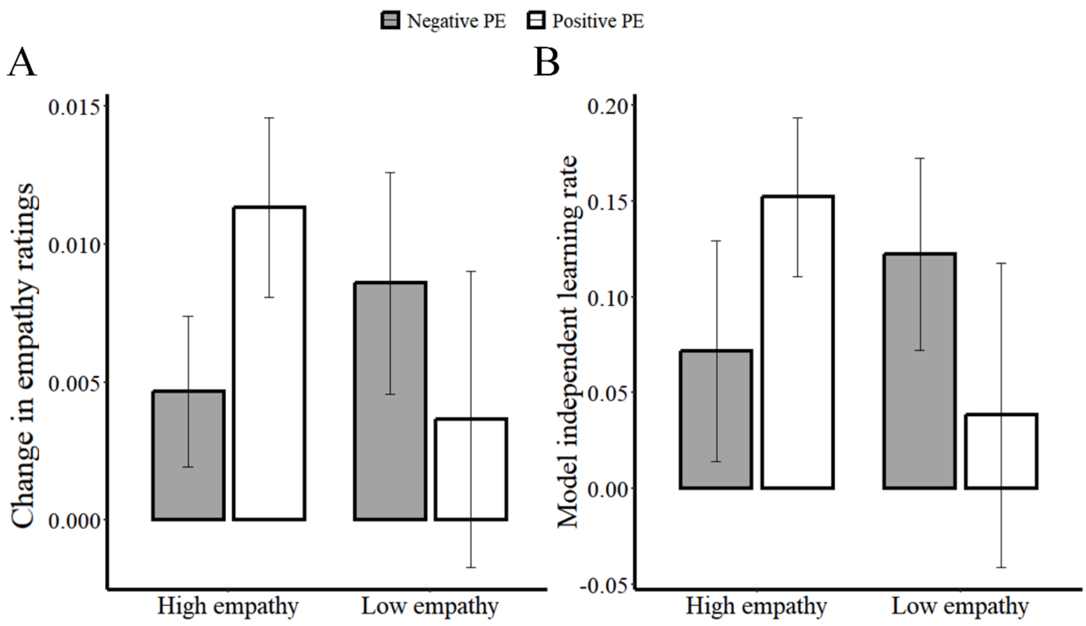

Figure S5. Changes in A) empathy ratings and B) model-independent learning rates in the high and the low empathy groups depending on different types of prediction errors (PE).

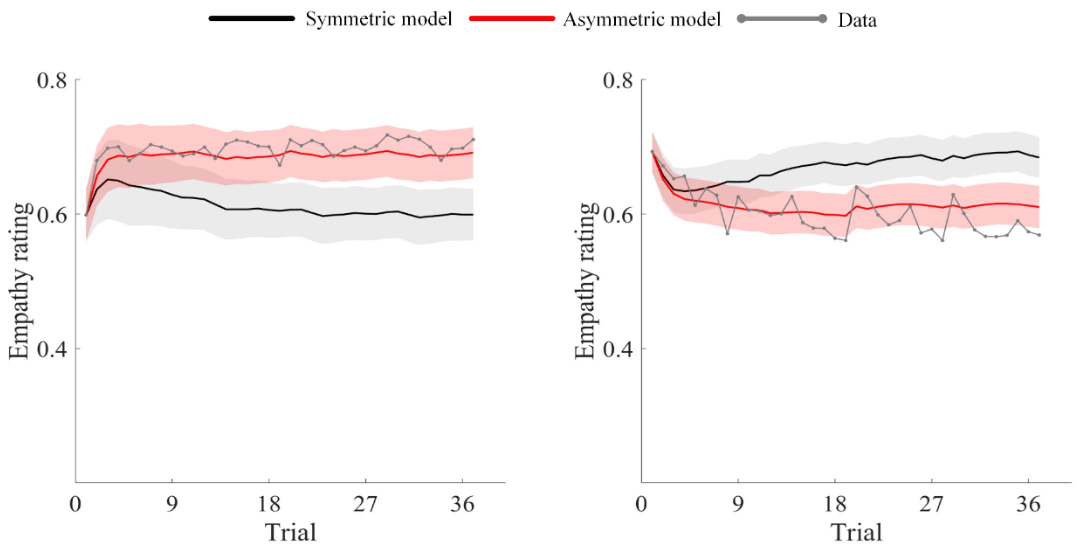

Figure S6. Simulation analyses. Trial-by-trial empathy ratings (grey line) and corresponding model estimates for the high and low empathy groups in the simulation analyses with symmetric (black line) or asymmetric learning (red line).

Supplementary Tables

Table S1. Predicting change in empathy ratings from baseline ratings, social desirability, and conformity.

ΔEmpathy ratings			
**Predictors**	**β (SE)**	**T-value**	**P-value**
High empathy group			
Baseline rating	0.096 (0.063)	0.43	0.675
Conformity	0.145 (0.075)	0.66	0.515
Social desirability	-0.219 (0.321)	-1.04	0.308
Low empathy group			
Baseline rating	0.126 (0.122)	0.59	0.558
Conformity	-0.158 (0.205)	-0.74	0.469
Social desirability	0.091 (0.677)	0.41	0.683

Table S2. Means and standard deviations for computational model parameters of the winning model for high and low empathy condition in the fMRI study.

**Computational Parameter**	**High empathy**	**Low empathy**
$W1_{pos}$	0.46 (0.35)	-0.18 (0.49)
$W2_{pos}$	0.41 (0.34)	-0.03 (0.37)
$W1_{neg}$	-0.10 (0.38)	0.25 (0.39)
$W2_{neg}$	-0.18 (0.47)	0.31 (0.36)
Forgetting parameter (γ)	0.80 (0.27)	0.90 (0.14)
Learning rate (α)	0.46 (0.14)	0.47 (0.13)

Note: The parameters *W1* and *W2,* which capture the weight of the influence of observational prediction errors on changes in participants' empathy ratings in the first/second half of the observational learning session.

Table S3: Brain regions correlating with trial-by-trial observational prediction errors for the high and the low empathy group separately and across both groups.

Region	Cluster Size	MNI Coordinates X	Y	Z	Peak z
**A) High empathy group**					
dmPFC	80	-6	48	34	3.90
**B) Low empathy group**					
R_premotor	122	52	4	48	4.93
L_fusiform	173	-44	-66	-24	4.68
Cerebellum	125	-8	-60	-12	4.68
L_premotor	82	-42	6	46	4.66
Precuneus	672	22	-74	44	4.50
Cerebellum	120	0	-70	-32	4.43
R_Lingual	86	10	-64	-12	4.41
dMCC/SMA	310	6	2	64	4.37
R_AI	82	46	2	2	4.27
Cuneus	551	2	-80	18	4.22
**C) High vs. low empathy groups**					
dMCC	316	4	4	64	5.01
R_Premotor	284	56	2	44	4.73
L_AI	280	-52	8	-12	4.73
L_Occipital	96	-46	-78	18	4.60
L_TPJ	192	-56	-58	20	4.45

dmPFC: dorsal medial prefrontal cortex. dMCC: dorsal medial cingulate cortex. SMA: Supplementary motor area. AI: anterior insula. TPJ: temporoparietal conjunction. L: Left, R: Right. Significant clusters were identified by combining a voxel-level threshold of $p < .001$ (uncorrected) and a cluster-level threshold of $p < .05$, FWE corrected.

Table S4. Results of linear mixed models predicting the change of empathy rating and differences across studies.

Study	Regressors	Statistic value χ^2	Statistic value p
**fMRI & Non-social control**	Empathy Group	1.24	0.26
	Prediction error	27.70	< 0.001
	Study	0.51	0.48
	Empathy Group ×Prediction error	0.55	0.46
	Empathy Group ×Study	0.0002	0.98
	Prediction error ×Study	5.34	0.021
	Empathy Group ×Prediction error ×Study	0.29	0.59
**fMRI & Behavioral replication**	Empathy Group	0.22	0.64
	Prediction error	53.62	< 0.001
	Study	0.0001	0.97
	Empathy Group ×Prediction error	0.25	0.62
	Empathy Group ×Study	0.50	0.48
	Prediction error ×Study	0.55	0.46
	Empathy Group ×Prediction error × Study	0.06	0.81
**fMRI & Behavioral replication & Behavioral without prediction**	Empathy Group	0.37	0.54
	Prediction error	65.75	< 0.001
	Study	0.25	0.88
	Empathy Group ×Prediction error	0.96	0.33
	Empathy Group × Study	0.46	0.80
	Prediction error ×Study	0.59	0.75
	Empathy Group × Prediction error × Study	0.34	0.84
**fMRI & Behavioral replication & Caucasian sample**	Empathy Group	0.44	0.51
	Prediction error	62.84	< 0.001
	Study	0.00	1.00
	Empathy Group × Prediction error	0.27	0.60
	Empathy Group × Study	0.57	0.75
	Prediction error × Study	2.17	0.34
	Empathy Group ×Prediction error × Study	0.08	0.96

Table S5. Results of linear mixed models testing the effects of empathy group (high/low), valence of prediction errors (positive/negative) and its interactions on changes in empathy ratings and model-independent learning rates.

Dependent variables	Regressors	Statistic value	
		χ^2	p
**Changes in empathy ratings**	Empathy Group	0.13	0.73
	Valence	0.28	0.60
	Empathy Group × Valence	12.01	< 0.001
**Learning rates**	Empathy Group	0.06	0.80
	Valence	0.00	1.00
	Empathy Group × Valence	5.39	0.02

Table S6. Results of questionnaire and behavioral measures within studies.

	Variables	High empathy group	Low empathy group	T test	
		Mean ± SD	Mean ± SD	*T-value*	*P*
Study 1	Age	21.1 ± 2.3	20.8 ± 1.8	-0.066	0.948
	Education	15.6 ± 2.0	15.5 ± 1.5	0.318	0.752
	IRI	97.6 ± 11.3	95.6 ± 9.3	0.698	0.488
	Contagion	23.4 ± 4.2	21.7 ± 3.4	1.572	0.122
	Empathy	24.3 ± 5.0	22.3 ± 3.7	1.630	0.109
	SDS	9.8 ± 3.7	10.5 ± 2.8	-0.720	0.475
	Conformity	56.5 ± 10.9	51.3 ± 9.1	1.862	0.068
Study 2	Age	20.7 ± 2.2	20.9 ± 2.6	-0.310	0.758
	Education	15.4 ± 2.3	15.4± 2.1	0.074	0.941
	IRI	97.7 ± 10.6	96.8 ± 9.6	0.356	0.723
	Contagion	22.4 ± 3.7	22.3 ± 3.4	0.118	0.907
	Empathy	22.1 ± 4.2	22.3 ± 3.8	-0.185	0.854
	SDS	8.9 ± 2.7	9.3 ± 2.8	-0.570	0.571
	Conformity	51.3 ± 12.9	56. 4 ± 12.3	-1.516	0.135
Study 3	Age	21.0 ± 1.9	20.5 ± 1.9	0.962	0.341
	Education	15.3 ± 1.7	14.7 ± 1.7	1.263	0.213
	IRI	99.6 ± 10.9	98.7 ± 13.0	0.280	0.780
	Contagion	22.4 ± 2.8	23.6 ± 4.4	-1.206	0.234
	Empathy	22.2 ± 2.8	23.0 ± 5.4	-0.658	0.514
	SDS	8.8 ± 3.1	9.6 ± 3.0	-0.981	0.331
	Conformity	53.0 ± 15.2	50.0 ± 12.0	0.805	0.425
Study 4	Age	21.3 ± 2.2	20.6 ± 2.2	1.241	0.220
	Education	16.0 ± 2.0	15.3 ± 1.8	1.354	0.182
	IRI	97.8 ± 9.6	98.6 ± 9.1	-0.336	0.738
	Contagion	22.9 ± 4.0	23.5 ± 3.2	-0.563	0.576
	Empathy	22.1 ± 4.5	23.3 ± 4.2	-0.942	0.351
	SDS	8.9 ± 3.0	8.7 ± 3.1	0.178	0.860
	Conformity	58.7 ± 12.1	55.4 ± 13.0	0.978	0.333

Education = Previous years of education; IRI = Interpersonal Reactivity Index (measure of trait empathy); SDS = Social Desirability Scale; Contagion = Behavioral Contagion; Empathy = Empathy Index.

SI References

1. X. Han, *et al.*, Cognitive and neural bases of decision-making causing civilian casualties during intergroup conflict. *Nat. Hum. Behav.* **5**, 1214–1225 (2021).

2. G. Hein, J. B. Engelmann, M. C. Vollberg, P. N. Tobler, How learning shapes the empathic brain. *Proc. Natl. Acad. Sci.* **113**, 80–85 (2016).

3. G. Hein, Y. Morishima, S. Leiberg, S. Sul, E. Fehr, The brain's functional network architecture reveals human motives. *Science* **351**, 1074–1078 (2016).

4. R. B. Rutledge, N. Skandali, P. Dayan, R. J. Dolan, A computational and neural model of momentary subjective well-being. *Proc. Natl. Acad. Sci.* **111**, 12252–12257 (2014).

5. G.-J. Will, R. B. Rutledge, M. Moutoussis, R. J. Dolan, Neural and computational processes underlying dynamic changes in self-esteem. *Elife* **6**, e28098 (2017).

6. Y. Zhou, *et al.*, Learning from ingroup experiences changes intergroup impressions. *J. Neurosci.* **42**, 6931–6945 (2022).

7. A. R. Wagner, R. A. Rescorla, Inhibition in Pavlovian conditioning: Application of a theory. *Inhib. Learn.*, 301–336 (1972).

8. N. D. Daw, S. J. Gershman, B. Seymour, P. Dayan, R. J. Dolan, Model-based influences on humans' choices and striatal prediction errors. *Neuron* **69**, 1204–1215 (2011).

9. A. Huebner, C. Wang, A Note on Comparing Examinee Classification Methods for Cognitive Diagnosis Models. *Educ. Psychol. Meas.* **71**, 407–419 (2011).

10. C.-C. Ting, S. Palminteri, M. Lebreton, J. B. Engelmann, The elusive effects of incidental anxiety on reinforcement-learning. *J. Exp. Psychol. Learn. Mem. Cogn.* **48**, 619 (2022).

11. J. Ashburner, K. J. Friston, Unified segmentation. *Neuroimage* **26**, 839–851 (2005).

12. K. J. Friston, *et al.*, Psychophysiological and modulatory interactions in neuroimaging. *Neuroimage* **6**, 218–229 (1997).

13. D. G. McLaren, M. L. Ries, G. Xu, S. C. Johnson, A generalized form of context-dependent psychophysiological interactions (gPPI): a comparison to standard approaches. *Neuroimage* **61**, 1277–1286 (2012).

14. K. C. Aberg, E. E. Kramer, S. Schwartz, Interplay between midbrain and dorsal anterior cingulate regions arbitrates lingering reward effects on memory encoding. *Nat. Commun.* **11**, 1829 (2020).

15. G. Lois, *et al.*, Large-scale network functional interactions during distraction and reappraisal in remitted bipolar and unipolar patients. *Bipolar Disord.* **19**, 487–495 (2017).

16. J. D. Power, *et al.*, Functional network organization of the human brain. *Neuron* **72**, 665–678 (2011).
